# Associations between Variability in Between- and Within-Day Dietary Intake with Adiposity and Glucose Homeostasis in Adults: A Systematic Review

**DOI:** 10.1016/j.advnut.2024.100310

**Published:** 2024-10-09

**Authors:** Perdana ST Suyoto, Nindya P Pamungkas, Jeanne HM de Vries, Edith JM Feskens

**Affiliations:** 1 Division of Human Nutrition and Health, Wageningen University and Research, Wageningen, the Netherlands; 2 Department of Nutrition and Health, Faculty of Medicine, Public Health and Nursing, Universitas Gadjah Mada, Indonesia

**Keywords:** adiposity, circadian disruption, dietary intake variability, glucose homeostasis, meal timing

## Abstract

This systematic review aims to comprehensively evaluate the literature regarding the impact of variations in dietary intake, both between- and within-day, on adiposity and glucose metabolism. We included observational and experimental articles obtained from PubMed, Scopus, Cochrane Library, and gray literature until 9 October, 2023, evaluating the impact of between- or within-day variations in meal, energy, or macronutrient intake on these outcomes. Our focus was on adults aged ≥18 y, spanning both healthy individuals and those with type 2 diabetes mellitus (T2DM). Given the diverse range of exposures, treatments, and outcomes among the selected articles, we chose a qualitative synthesis approach to effectively analyze the data. Eighty articles from 43 observational and 37 experimental studies were included, involving 89,178 participants. Patterns of dietary intake variation were identified and systematically organized into distinct categories based on similarities. Between-day variations in dietary intake consisted of between-day variations in both the quantity consumed and meal timing. Meanwhile, within-day variations encompassed factors such as eating window, meal omission, within-day meal timing, within-day variation in dietary intake quantity, and temporal distribution. Despite mixed results, time-restricted eating was generally associated with lower adiposity. However, limited control for total daily energy intake (TDEI) suggests that the contribution of lower energy intake cannot be conclusively excluded. Conversely, the adverse effect of meal omission on glucose parameters was consistently supported by randomized trials. Interestingly, the results showed that consuming a substantial portion of TDEI in the morning may increase the likelihood of observing improvements in adiposity. Furthermore, inconsistencies in outcomes across articles examining the effects in healthy compared with T2DM populations, or in energy-sufficient compared with deficient individuals, indicate potential condition-specific effects. These findings support the need for further investigation into the effects of between- and within-day variations in dietary intake to better understand their impact on adiposity and glucose homeostasis.

This review was registered in PROSPERO as CRD42020214307.

**Statement of significance**

Our systematic review offers a novel and comprehensive analysis of how variations in dietary intake patterns, both between and within days, influence adiposity and glucose homeostasis. To advance future research, we critically examine the potential factors contributing to the observed inconsistencies across studies investigating these relationships.

## Introduction

It has been demonstrated using various study designs that adopting a healthy dietary pattern is associated with a reduced risk of obesity and type 2 diabetes mellitus (T2DM) [[1], [2], [3], [4], [5], [6]]. Although these findings are useful, dietary assessments in most studies often rely on the use of food frequency questionnaires or other self-reports, which limit the scope of dietary patterns to the type and quantity of food. Traditionally, dietary intake from several days of observations is averaged to obtain usual intake per day [7]. However, dietary intake does not only vary from day to day but also within each day. For example, variations in dietary intake may arise from differences in dietary intake between mealtimes within a single day. Both between-day and within-day variations in dietary intake, whether in timing or quantity, are becoming increasingly relevant, especially in research on the impact of meals on circadian rhythms, an area receiving more attention recently [8,9].

Meal timing, through its modulation of circadian rhythm, plays a significant role in relation to adiposity and glucose homeostasis. One underlying mechanism involves insulin, a time cue (*zeitgeber*) for the peripheral circadian clock [10]. Diet-induced insulin secretion indirectly affects the peripheral clock and subsequent downstream signaling pathways, including those in adipogenesis [11]. Consuming a meal at an atypical circadian time, such as during the biological rest phase, poses deleterious effects on glucose and fat metabolism. Feeding at the rest phase dampened clock gene expressions in adipose tissue, doubled plasma insulin levels, and increased body weight (BW) in rodents [12]. In concordance, human studies have shown that metabolism responds differently to meal consumption at different time points during the day. In the evening, the area under the curve (AUC) for postprandial glucose and insulin’s incremental area under the curve (iAUC) were higher than those in the morning after administration of the oral glucose tolerance test (OGTT) [13].

Similar to mistiming meals within days, growing evidence suggests that irregular between-day eating patterns could also adversely affect metabolism, specifically regarding the development of adiposity. Because the circadian clock is finely tuned to anticipate recurring environmental changes [14], maintaining a consistent environment may be vital to maintaining a healthy rhythm. A cross-sectional study showed that irregular timing of meals is associated with higher BMI in healthy young adults aged 18 to 25 y [15]. Moreover, irregular consumption of dietary intake may also lead to negative metabolic consequences, as shown by higher BW observed among individuals exhibiting irregular breakfast habits or inconsistent between-day energy intake [16,17].

Variability in dietary intake, both between days or within a single day, can be assessed in various ways. These include analyzing the distribution of energy and nutrient intake over the day, the percentage of nutrients consumed during specific mealtimes, categorization of subjects into early or late eaters according to predominant periods of energy or nutrient consumption, examining measures of the variability in energy or nutrient intake between days or within a day (between-meals), assessing eating window duration, and identifying instances of meal omission [[16], [17], [18], [19], [20], [21], [22]]. Therefore, the purpose of this systematic review was to summarize existing evidence on these forms of dietary intake variability, focusing on energy and macronutrient intake, and to evaluate their associations with the main risk factors for T2DM, adiposity, and glucose homeostasis.

## Methods

The present systematic review was reported using the PRISMA guidelines (Supplementary Tables 1 and 2) [23]. The protocol of this study was preregistered in PROSPERO (CRD42020214307). No substantial change to the protocol was made during the preparation of this systematic review.

### Data sources and searches

This systematic review accommodated observational and experimental articles although excluding reviews and nonhuman study articles. A comprehensive literature search was conducted across multiple electronic databases including PubMed, Scopus, and the Cochrane Library, as well as gray literature databases (Supplementary Table 3). In addition, efforts were made to obtain every published full text from conference abstracts identified during the search. Only original articles written in English were included in this systematic review. A cutoff date limitation was set for articles published until 9 October, 2023.

We collected published articles evaluating the association of between- or within-day variation in dietary intake with adiposity and glucose homeostasis in individuals aged ≥18 y, including both healthy individuals and those with T2DM. Articles involving pregnant participants and inpatients were excluded. Dietary data under investigation included energy and macronutrient intake derived from the consumption of both foods and beverages. Henceforth, we will refer to both foods and beverages collectively as “food.” Between-day variation in dietary intake corresponded to the variability in the quantity or timing of energy or macronutrient (defined as protein, fat, and carbohydrates) intake from one day to another. Within-day variation of dietary intake was defined by fluctuations in energy or macronutrient intake due to variations in timing, distribution, or omission within a given day. To qualify for selection, articles needed to provide information from multiple mealtimes to determine the between- or within-day variation in dietary intake. Therefore, articles with outcomes solely focused on the acute effects of single meal consumption or particular mealtime were excluded from our review. In our analysis, we categorize all principal eating occasions—breakfast, lunch, and dinner—as well as snacks, under the umbrella term “meals” to encompass the full spectrum of dietary intake.

Adiposity outcome variables studied included BW, BMI, body fat (BF), and waist (WC) and hip circumference. In the domain of glucose homeostasis, we included measurements of glucose and insulin sampled from various sources such as venous, arterial, and capillary blood, as well as from interstitial fluid as obtained from continuous glucose monitoring (CGM). We also considered indexes derived from these biomarkers, including indexes of insulin sensitivity and insulin secretory function.

The detailed search strategy and the search strings used for each database can be found in the Supplementary Table 3. Briefly, keywords used in the search mainly consisted of 3 parts: variation (variation, dissimilarity, similarity, evenness, variability, fluctuation, disequilibrium, inconsistency, consistency, inconsistent, consistent, irregularity, regularity, irregular, regular, and distribution), dietary intake (dietary intake, calorie, energy, nutrient, macronutrient, [simple and complex] carbohydrate, protein, and fat intake), and the outcomes (overweight, obesity, obese, body mass index, BMI, body fat, body fatness, fat percentage, body composition, and several glucose and insulin parameters). We refrained from using reference lists of articles to identify additional articles. This decision was made because articles with positive results tended to be cited more frequently than those with negative results, which could potentially introduce citation bias [24].

### Article selection process

Two reviewers (PS and NP) screened titles and abstracts. Metadata containing titles and abstracts from the aforementioned databases were downloaded and imported into the Rayyan app [25] to identify and manually remove duplicates. Subsequently, full texts were obtained and independently evaluated by 3 reviewers (PS, JV, and NP). The decision to include any of these articles in the present systematic review was made collectively by the 3 reviewers (PS, JV, and NP).

### Quality assessment

Two reviewers (PS and NP) conducted risk of bias assessments using specific instruments tailored to the study design. Cross-sectional and cohort articles were evaluated using the National Heart, Lung, and Blood Institute (NHLBI) Quality Assessment Tool [26], although nonrandomized and randomized trials were assessed using The Risk Of Bias In Non-randomized Studies of Interventions (ROBINS-I) assessment tool [27] and Cochrane risk-of-bias tool for randomized trials (RoB2) [28], respectively. The NHLBI tool consists of 14 questions for both cross-sectional and cohort articles. Quality assessments were performed by choosing between “Yes,” “No,” or “Others” (CD, cannot determine; NA, not applicable; NR, not reported). Ratings were determined based on the ratio of “Yes”: (‘Yes” + “No”) responses as follows: Good: >66.6%, Fair: 33.3-66.6%, Poor: <33.3%. In ROBINS-I, various questions were addressed to identify bias across 7 domains, including bias due to confounding, bias in participant selection, bias in intervention classification, bias due to deviations from intended interventions, bias due to missing data, bias in outcome measurement, and bias in the selection of reported results. An overall rating of “Low,” “Moderate,” “Serious,” “Critical,” or “No Information” was obtained from this evaluation process. RoB2 encompasses 5 bias domains, including the risk of bias arising from the randomization process, bias due to deviations from intended interventions (effect of assignment to intervention), missing outcome data, bias in outcome measurement, and bias in the selection of the reported result. The overall risk of bias in each article was determined by assessing the rating of each domain, resulting in an overall rating of “Low,” “High,” or “Some concerns.” A summary table of the risk of bias assessment from each article can be found in Supplementary Tables 4‒6.

### Data extraction, synthesis, and analysis

Data from the selected full-text articles, including author names, publication years, aims, study designs, sample sizes, subjects’ characteristics, and findings, were extracted and organized in a spreadsheet file. We reported several effect measures, including mean differences, prevalence rates, correlation coefficients, and measures of risk, such as odds ratio (OR), relative risk ratio, and hazard ratio. Efforts were made to combine similar exposures or treatments into comparable categories to facilitate between-article comparison. However, due to the considerable variations in methods, exposures, or treatments and the analysis of outcomes among selected articles, a meta-analysis could not be performed. Consequently, all articles were included in the qualitative synthesis, with data presented in tabular form.

## Results

### Article selection

**FIGURE 1:**
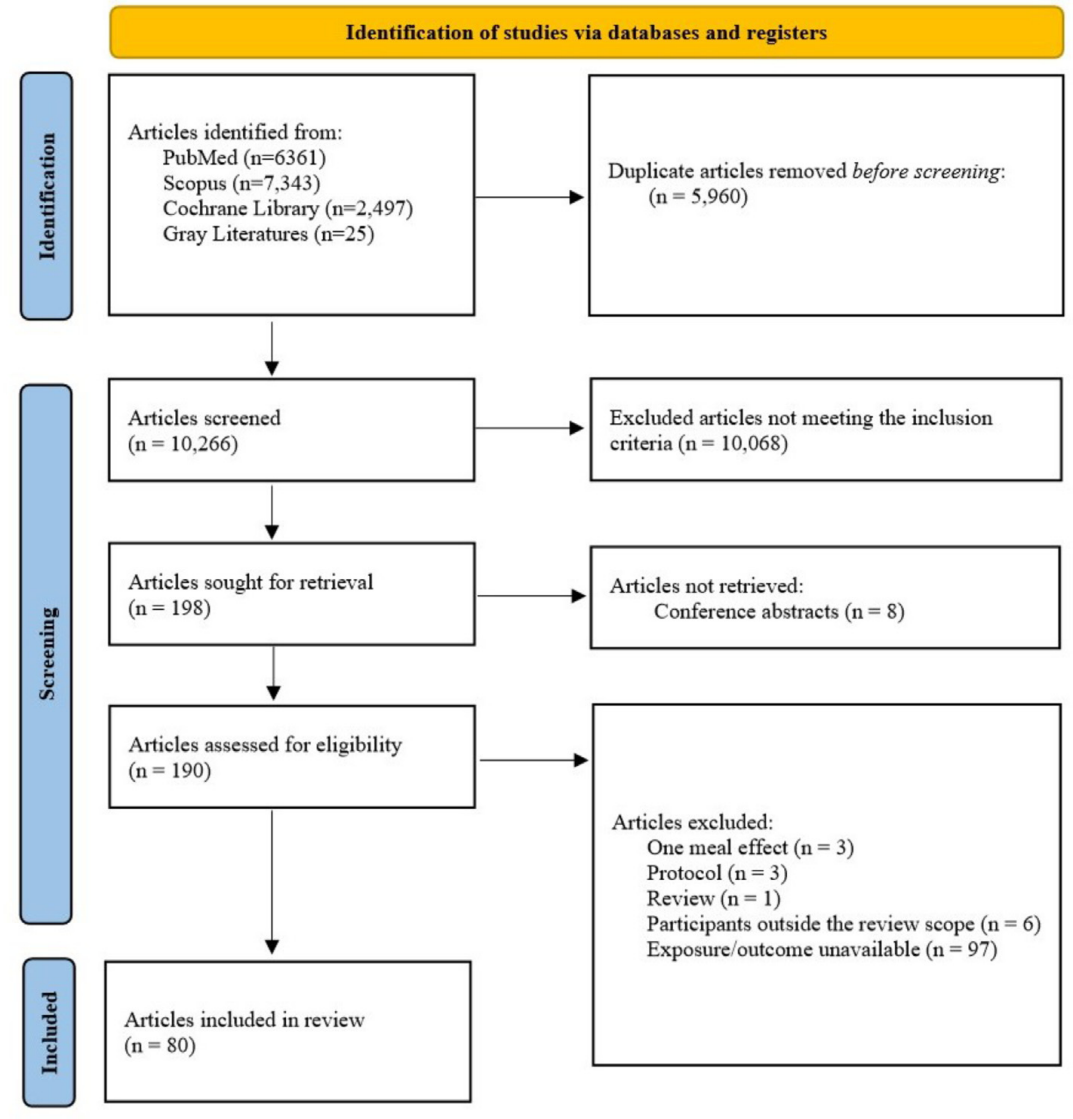
PRISMA flow diagram of included articles.

An initial electronic database search yielded 16,226 titles (Figure 1). Following the removal of 5960 duplicates, the remaining 10,266 titles underwent abstract-level screening. During this phase, 10,068 abstracts were excluded due to their lack of relevance to the review topic, and the full texts of the 198 articles deemed relevant were retrieved. However, full texts from 8 conference abstracts were not found. One hundred ten articles were excluded for various reasons. Ultimately, 80 articles that met the inclusion criteria were included in this review. The included studies consisted of 34 cross-sectional articles, 9 cohort articles, 6 articles with a one-group pretest-posttest design, 1 article with a nonrandomized parallel design, 16 articles with a randomized parallel-design, and 14 crossover trials involving a total of 89,178 participants. The included articles were then categorized based on similarities in exposures or treatments, focusing on variations in dietary intake between days and within a single day.

Between-day variation in dietary intake consists of fluctuations in both the quantity and timing of meals across different days. The variation in dietary intake quantity between days refers to differences in the amount of energy, protein, fat, or carbohydrate consumed; in addition, between-day variation in meal timing refers to differences in the timing of meals across days including the clock time at which particular meals, such as breakfast, lunch, or dinner, occur as well as any representations of the entire daily meal, such as caloric midpoint.

**FIGURE 2:**
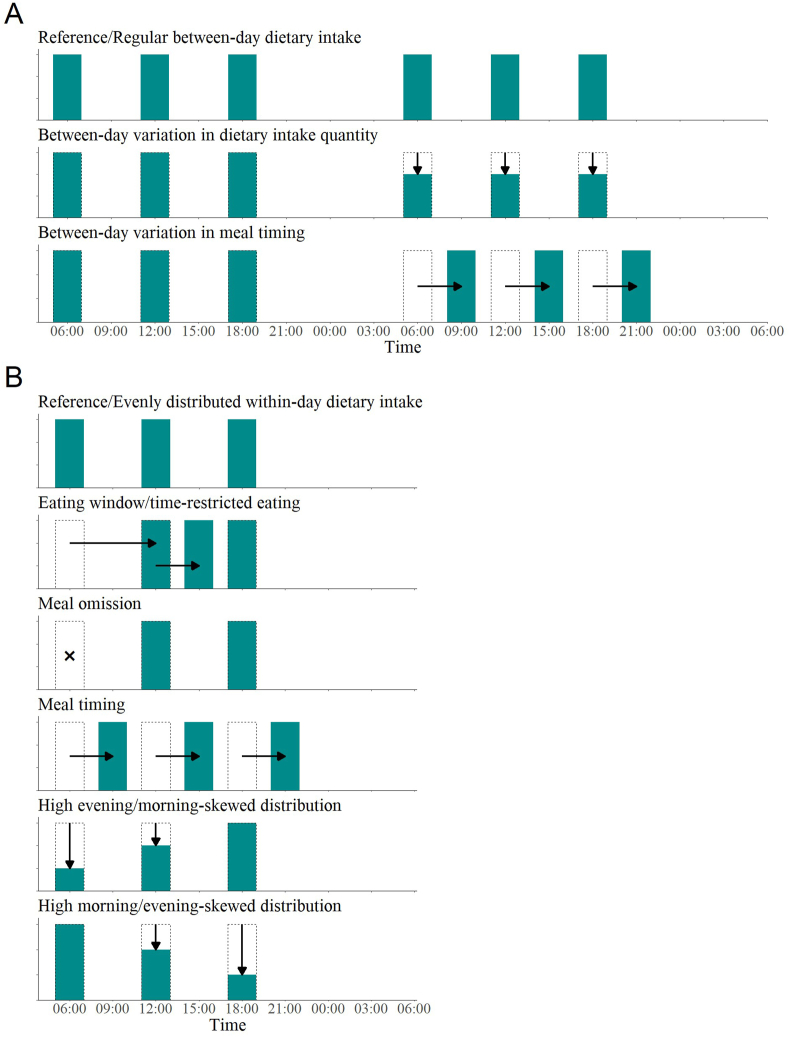
Graphical description of between-day (A) and within-day (B) dietary intake variation. The bars’ heights represent the quantity of energy or nutrient intake, and their positions on the horizontal axis explain the timing of meal events. Between-day variation in dietary intake quantity is characterized by the difference in the amount of energy or macronutrient intake from one day to the other. In the between-day variation of meal timing, the difference lies in the meal consumption time, depicted as a shift in the meal events. In within-day dietary intake variation in meal timing, the difference in meal timing occurs between- not within-person. Meal omission is described as the absence of one meal consumption although time-restricted eating aggregates all dietary intake within a shorter period of time compared to the reference. Morning- and evening-skewed distribution of dietary intake is illustrated by a lower quantity of intake in the morning and evening, respectively, which explains variations in between-day dietary intake quantity and temporal distribution of dietary intake. Bars with dashed borders depict reference dietary intake (either regular between-day or evenly distributed within-day dietary intake) to facilitate visual assessment of dietary intake variability. The changes from reference in terms of quantity or timing are depicted by arrows. Cross (×) sign indicates the absence of a mealtime.

Within-day variation in dietary intake included eating window, meal omission, and meal timing, as well as variations in dietary intake quantity and temporal distribution within a single day. The term eating window refers to the clock time interval during which an individual consumes food. The dietary strategy of narrowing this eating window to shorter time intervals is known as time-restricted eating (TRE). Meal omission refers to the act of skipping ≥1 mealtimes. Within-day variation in dietary intake quantity explains the fluctuations in energy or macronutrients consumption between mealtimes within a single day. The temporal distribution elucidates how energy or macronutrient intake is distributed throughout the day. This distribution can be uniform across mealtimes, skewed toward the evening with higher intake in the morning, or skewed toward the morning with lower intake in the evening. Graphical representations illustrating these dietary intake variations in both between-day and within-day contexts are presented in Figure 2.

### Risk of bias analysis

The risk of bias assessment revealed an overall low risk of bias in half of the cross-sectional and cohort articles, as well as in all nonrandomized intervention articles. Sixteen of 30 randomized trials were rated as having ‘some concerns,” although none were considered to have an overall high risk of bias.

Only 5 of 43 observational articles provided justifications for their sample size or power calculations. In one nonrandomized parallel-design article [29], researchers attempted to control for baseline confounders by matching the experimental groups for sex, age, BMI, and physical activity. Nevertheless, inherent to this type of study is the potential bias arising from residual confounding.

The majority of included randomized trials raised concerns about potential bias stemming from the randomization process because it was not clear whether allocation concealment was performed. Lack of blinding was also prevalent in the intervention studies but cannot be avoided given the nature of the dietary treatment. One trial [30] reported making the dietary treatments indistinguishable in an effort to minimize this bias. Lastly, a small number of both randomized (*n* = 6) [[31], [32], [33], [34], [35], [36]] and nonrandomized (*n* = 1) [37] trials lacked information on retrospective protocol registration. This absence of registration may contribute to potential bias in the selection of reported results.

The results were structured into 7 distinct dietary intake variations. These began with between-day differences, which covered variations in both dietary intake quantity and meal timing. The analysis then progressed to within-day differences, examining factors such as eating windows, meal omission, meal timing, within-day variation in dietary intake quantity, and temporal distribution. For each dietary intake variation, we classified the findings based on their associations with adiposity or glucose homeostasis.

### Between-day variation in dietary intake quantity

#### Adiposity

Four cross-sectional articles on this topic reported mixed findings regarding the association between variability in energy intake and adiposity (Table 1). Two articles (*n* = 73‒7958) observed positive associations between these variables, indicating an increased risk of general (adjusted OR: 1.24; 95% confidence interval [CI]: 0.95, 1.61) and central obesity (adjusted OR: 1.22; 95% CI: 1.03, 1.43) [38] as well as BMI (*r* = 0.36; *P* = 0.01) and WC (*r* = 0.28; *P* = 0.04) [17], although the other 2 articles (*n* = 259‒850) reported no such association [39,40].

**TABLE 1:** Relationship of between-day variation in dietary intake quantity with adiposity and glucose homeostasis.

Authors	Primary aim(s)	RoB	Design, follow-up period	Definition of meal variation	Intervention/grouping	*n*	Participants	Findings on adiposity	Findings on glucose homeostasis
Saneei et al., 2016 [38]	To investigate the relationship between dietary habit patterns, identified by LCA, and obesity.	Fair	Cross-sectional	‘Irregular meal pattern’ derived from LCA	—	7958	Men and women (60% women)	Irregular vs. regular meal pattern (adjusted for covariates): - increased obesity prevalence by 2.2% - increased general and central obesity risk by >20%	—
Terada et al., 2019 [17]	To evaluate the association between snacking frequencies, fasting duration, and between-day energy intake variability with cardiometabolic and psychological health in female nurses.	Fair	Cross-sectional	CV of between-day energy intake	—	73	Female nurses aged (mean ± SD) 46.6 ± 10.8 y	CV of energy intake: - correlated with BMI, *r* = 0.356 & WC, *r* = 0.283 - not correlated with %BF	—
Jayedi et al., 2020 [39]	To investigate the association of daily irregularity in energy intake with diet quality among healthy adults.	Fair	Cross-sectional	Irregularity score derived from the difference between daily energy intake and 3-d mean energy intake divided by the 3-d mean energy intake, multiplied by 100, and mean averaged	—	850	Men (*n* = 266) and women (*n* = 583) aged 20–59 y	T3 vs. T1 of irregularity score: - no difference in BMI and WC	—
Park et al., 2020 [40]	To classify the dietary behaviors of obese and overweight participants and to evaluate the association between dietary behavior patterns and cardiometabolic risk factors.	Fair	Cross-sectional	‘Irregular unhealthy eating behavior” derived from LCA	‘Healthy but unbalanced” eaters (*n* = 118); or ‘Irregular, unhealthy” eaters (*n* = 88)	259	Men (*n* = 163) and women (*n* = 96) aged ≥20 y	‘Irregular, unhealthy” vs. “healthy but unbalanced” (adjusted for covariates): - reduced BMI by 2.7 kg/m^2^, WC by 4 cm, and %BF by 6.9%	‘Irregular, unhealthy” vs. “healthy but unbalanced” (adjusted for covariates): - no difference in fasting glucose
Pot et al., 2016 [41]	To evaluate the associations of irregular meal energy intake with cardiometabolic risk factors after 10 and 17 y.	Good	Cohort, 17 y	Irregularity score derived from the deviation of energy intake of a mealtime from 5-d mean energy intake of that mealtime	—	1416	Men (*n* = 666) and women (*n* = 750) aged 36 y	T3 vs. T1 irregularity score of energy intake at the age of 36y (adjusted for covariates): - increased central obesity risk by 40% and overweight risk by 34% 17 y later	T3 vs. T1 irregularity score of energy intake at the age of 36 y (adjusted for covariates): - difference in HbA1c
Eom et al., 2022 [42]	To investigate the relationship between meal irregularity and weight-loss.	Good	Cohort, 45.7 d	Between-day variation in energy intake	Regular energy intake; or Irregular energy intake	637	Women aged (mean ± SD) 33.2 ± 7.2y with BMI of (mean ± SD) 25.6 ± 3.6 kg/m^2^	Regular vs. irregular energy intake: - increased weight loss by 1 kg	—
Bowen et al., 2018 [43]	To compare the effect of a high protein meal that provides the same daily energy restriction vs. alternate energy restriction consisting of 3 d of alternate-day modified fasting, 3 d of alternate daily energy restriction, and one ad libitum day on weight loss and body composition in overweight and obese adults.	Some concerns	Randomized parallel-design, 16 wk	Between-day variation in energy intake	DER: a high protein, meal replacement program with daily energy restriction (*n* = 68); or ADF + DER: alternating days of modified fasting and DER plus 1 ad libitum d/wk (*n* = 67)	135	Overweight/obese men (*n* = 30) and women (*n* = 132) aged (mean ± SD) 40 ± 8 y	DER vs. ADF+DER: - no difference in BMI and BF	DER vs. ADF + DER: - no difference in fasting glucose and fasting insulin
Beaulieu et al., 2020 [44]	To assess the effects of continuous and intermittent energy restriction on appetite.	Low risk	Randomized parallel-design, 12 wk	Between-day variation in energy intake	Continuous energy restriction (*n* = 19); or Intermittent energy restriction (*n* = 18)	37	Overweight and obese women aged 18–55 y	Continuous vs. intermittent energy restriction: - no difference in BW, BMI, BF, %BF, WC, HC, and WHR	—

Abbreviations: ADF, alternate-day modified fasting; BF, body fat; BMI, body mass index; BW, body weight; CV, coefficient of variation; DER, daily energy restriction; HbA1c, glycated hemoglobin; HC, hip circumference; LCA, latent class analysis; RoB, risk of bias; SD, standard deviation; WC, waist circumference; WHR, waist-hip ratio.

In contrast to the cross-sectional articles, 2 cohort articles rated as “good” in the risk of bias analysis showed consistent results. A cohort study involving 1416 participants [41] provided evidence for a positive association between between-day variation in the amount of energy intake and adiposity. The study utilized irregularity scores based on the variation in energy intake at specific mealtimes. Individuals with higher irregularity scores (tertile 3 compared with 1) at age 36 y exhibited a significantly increased risk of central obesity (adjusted OR: 1.40; 95% CI: 1.13, 1.73) and overweight (adjusted OR: 1.34; 95% CI :1.05, 1.72) at age 53 y. Furthermore, participants with higher irregularity scores of energy at breakfast and lunch had a >50% increased risk of overweight and central obesity. Similarly, the other cohort article (*n* = 637) [42] reported a protective association between regularity in energy intake between days and BW reduction. It is essential to note that this study did not adjust for the impact of physical activity and total daily energy intake (TDEI). Therefore, the potential impact of these factors on the observed results remains unknown.

Despite relatively long follow-up periods (12–16 wk), the 2 randomized trials included (*n* = 37‒135) failed to demonstrate a significant effect of between-day variation in energy intake on adiposity measures [43,44]. These articles employed an alternate-day dietary protocol in which participants consumed an energy-restricted diet on one day and one with 48% [43] or 25% [44] fewer calories on the other day.

The lack of effects observed in randomized trials with controlled energy intake suggests minimal influence of between-day energy intake variations on adiposity. This stands in contrast with the somewhat positive findings from the cohort articles. However, the limitations of observational studies, including potential confounding by uncontrolled variables such as TDEI and physical activity, underscore the need for further investigation with robust study designs to draw definitive conclusions.

#### Glucose homeostasis

No associations were observed between between-day variation in energy intake and glucose homeostasis in either observational (*n* = 259‒1,416) [40,41] or randomized trials (*n* = 135) [43], even though such intake was associated with reduced adiposity (Table 1). Glucose parameters assessed in these studies were fasting glucose [40,43], fasting insulin (43), and glycated hemoglobin (HbA1c) [41].

The absence of effects in the included articles within this category could be attributed to limited statistical power due to small sample sizes or to insufficient control for potential confounding factors such as physical activity and dietary composition. Consequently, based on the current evidence, we cannot conclusively determine whether between-day energy intake variations influence glucose homeostasis.

### Between-day variation in meal timing

#### Adiposity

Four cross-sectional studies employed different measures to describe between-day variation in meal timing, including SD of the first and last meal timing (*n* = 73) [45], irregular mealtime score (*n* = 12,017) [46], and eating jet lag, which is the difference in meal timing between weekends and weekdays, and adiposity (*n* = 118‒1106) [15,47] (Table 2). A small cross-sectional study showed that a higher SD of the time of first meal consumption between days, indicating irregular first meal timing, was associated with increased body fat (β: 0.23 kg; 95% CI: 0.02, 0.43 kg; *P* = 0.03) after adjusting for confounding factors [45]. Additionally, participants in the fourth quartile (Q4) with a higher irregular mealtime score, as determined by factor analysis, compared to those in the first quartile (Q1), exhibited a higher age- and sex-adjusted BMI by 0.6 kg/m^2^ (Q4 compared with Q1 (mean ± SD): 22.9 ± 0.1 compared with 22.3 ± 0.1 kg/m^2^; *P* < 0.001) and a larger age- and sex-adjusted visceral fat area by 8 cm^2^ (Q4 compared with Q1 (mean ± SD): 76 ± 1 compared with 68 ± 1 cm^2^; *P* < 0.001) [46]. Contradictory findings emerged between 2 studies [15,47] regarding the association between eating jet lag. A smaller study reported no association [47], whereas a larger study found that a 0.28-h (95% CI: 0.080, 0.479) difference in meal timing between weekdays and weekends (eating jet lag) was associated with a BMI increase of 1 kg/m^2^ [15].

**TABLE 2:** Relationship of between-day variation in meal timing with adiposity and glucose homeostasis.

Authors	Primary aim(s)	RoB	Design, follow-up period	Definition of meal variation	Intervention/grouping	*n*	Participants	Findings on adiposity	Findings on glucose homeostasis
Takase et al., 2019 [46]	To investigate the association between dietary factors and visceral fat accumulation.	Fair	Cross-sectional	Dietary habit of “irregular mealtime” derived from factor analysis with varimax rotation	—	12,017	Men (*n* = 8746) and women (*n* = 3271) aged >20 y	Q4 vs. Q1 irregular mealtime score (adjusted for age and sex): - increased BMI by 0.6 kg/m^2^ & VF area by 8 cm^2^	—
Zerón-Rugerio et al., 2019 [15]	To investigate the association between high variability in meal timing on weekends compared with weekdays and obesity.	Fair	Cross-sectional	Eating jet lag: the discrepancy between meal timing on weekends and weekdays	—	1106	Men aged 18–25 y	Eating jet lag of 0.28 h (adjusted for covariates): - increased BMI by 1 kg/m^2^	—
Zhao et al., 2022 [45]	To investigate the relationship between eating architecture and body fat and markers of glycemic control in adults at increased risk of T2DM.	Good	Cross-sectional	Irregularity of the first and last meal timing	—	73	Men (n = 39) and women (n = 34) aged (mean ± SD) 58.8 ± 8.1 y	1 SD of irregular first meal timing (adjusted for covariates): - increased BF by 0.23 kg Irregular last meal timing (adjusted for covariates): - no difference in BF	1 SD of irregular first meal timing (adjusted for covariates): - no difference in HbA1c Irregular last meal timing (adjusted for covariates): - no difference in HbA1c
Dote-Montero et al., 2023 [47]	To investigate the association of meal timing with body composition and cardiometabolic risk factors in young adults.	Fair	Cross-sectional	Eating jet lag	—	118	Men (*n* = 36) and women (*n* = 82) aged (mean ± SD) 22 ± 2y	Eating jet lag (adjusted for covariates): - no association with BMI, BF, and WC	Eating jet lag was associated with (adjusted for covariates): - no association with HOMA-IR
Eom et al., 2022 [42]	To investigate the relationship between meal irregularity and weight loss.	Good	Cohort, 45.7 d	Between-day variation in meal timing	Regular mealtime; or Irregular mealtime	637	Women aged (mean ± SD) 33.2 ± 7.2 y with BMI of (mean ± SD) 25.6 ± 3.6 kg/m^2^	Regular vs. irregular mealtime: - increased weight loss by 0.87 kg	—

Abbreviations: BMI, body mass index; BF, body fat; HbA1c, glycated hemoglobin; HOMA-IR, homeostasis model assessment of insulin resistance; RoB, risk of bias; SD, standard deviation; VF, visceral fat; WC, waist circumference.

The association between variation in meal timing between days and adiposity was further supported by a high-quality cohort study (*n* = 637) that showed a lower coefficient of variation (CV) in breakfast, lunch, and dinner timing was associated with enhanced weight loss (breakfast: β: −0.091, *P* < 0.001; lunch: β: −0.053, *P* = 0.035; dinner: β: −0.058, *P* = 0.023) [42]. Although this study adjusted for various potential confounders such as age, BMI, and dietary intake, it lacked control for physical activity, which may have influenced the observed results.

As randomized trials are lacking in this research area, establishing a causal relationship between between-day variation in meal timing and adiposity is currently not possible. However, the 5 included observational articles appear to concur on the association between between-day variation in meal timing and adiposity. This evidence warrants further investigation to solidify the potential link and understand the underlying mechanisms.

#### Glucose homeostasis

Two studies investigated the association between between-day variation in meal timing, as indicated by the SD of first and last meal timing (*n* = 73) [45] and eating jet lag (*n* = 118) with glucose parameters [47] (Table 2). Regarding glucose homeostasis, between-day variation in both first and last meal timing was not significantly associated with HbA1c, a measure of long-term blood glucose control [45]. Similarly, eating jet lag was not linked to HOMA-IR, an index of insulin resistance [47].

Given the scarcity of research on this topic, drawing definitive conclusions and gaining insights regarding the association between between-day meal timing variations and glucose homeostasis remains premature. Therefore, we advocate further investigations to elucidate this potential relationship.

### Eating window

#### Adiposity

Two cross-sectional studies assessed the relationship between the eating window and various measures of adiposity (Table 3). One study measured BF (*n* = 73) [45], although the other measured BF, BMI, and WC (*n* = 118) [47]. However, neither of these studies revealed significant associations between the eating window and any of the measures of adiposity mentioned [45,47].

**TABLE 3:** Relationship of eating window with adiposity and glucose homeostasis.

Authors	Primary aim(s)	RoB	Design, follow-up period	Definition of meal variation	Intervention/grouping	*n*	Participants	Findings on adiposity	Findings on glucose homeostasis
Zhao et al., 2022 [45]	To investigate the relationship between eating architecture and body fat and markers of glycemic control in adults at increased risk of T2DM.	Good	Cross-sectional	Duration of eating window	—	73	Men (*n* = 39) and women (*n* = 34) aged (mean ± SD) 58.8 ± 8.1 y	Eating window duration: - no difference in BF	Eating window duration: - no difference in HbA1c
Dote-Montero et al., 2023 [47]	To investigate the association of meal timing with body composition and cardiometabolic risk factors in young adults.	Fair	Cross-sectional	Duration of eating window	—	118	Men (*n* = 36) and women (*n* = 82) aged (mean ± SD) 22 ± 2 y	Eating window (adjusted for covariates): - no association with BMI, BF, or WC	Eating window was associated with (adjusted for covariates): - inversely associated with HOMA-IR
Yoshimura et al., 2023 [48]	To investigate if an individual’s day-to-day nutrition-related lifestyle behaviors (meal timing, eating window, food intake, movement behaviors, sleep conditions, and body weight) impact daily glycemic outcomes under free-living conditions.	Fair	Cross-sectional	Duration of eating window	—	104	Men (*n* = 44) and women (*n* =60) aged (mean ± SD) 40 ± 12 y	—	Eating window was associated with (in mg/dL): - associated with mean (β = 9.49), SD (β = 11.8), and peak glucose (β = 46.7) - correlated with SD glucose (*r* = 2.48) - not correlated with mean or peak glucose level
Gill and Panda, 2015 [37]	To test the influence of reducing the daily eating window from >14 h to 10–11 h on the body weight of overweight and obese participants.	Low risk	One-group design, 16 wk	Duration of eating window	—	8	Overweight men (*n* = 5) and women (*n* = 3) aged 27.6 y (95% CI: 26.4, 28.8)	10 h TRE: - reduced BW by 3 kg	—
Wilkinson et al., 2020 [49]	To assess the effect of time-restricted feeding on cardiometabolic health in subjects with metabolic syndrome.	Low risk	One-group design, 12 wk	Duration of eating window	—	19	Men (*n* = 13) and women (*n* = 6) aged (mean ± SD) 59 ± 11 y with metabolic syndrome	10 h TRE: - reduced BW by 3.3 kg, BMI by 1 kg/m^2^, %BF by 1%, and WC by 4.46 cm	10 h TRE: - no difference in fasting glucose, insulin, HbA1c, and HOMA-IR
Parr et al., 2020 [50]	To determine the feasibility of TRE for individuals with T2DM.	Low risk	One-group design, 4 wk	Duration of eating window	—	19	T2DM men (*n* = 9) and women (*n* = 10) aged (mean ± SD) 50 ± 9 y	9 h TRE (10:00–19:00): - no difference in BW, BF	9 h TRE (10:00–19:00): - no difference in fasting HbA1c, glucose, and insulin - no difference in AUC glucose and insulin after meal tolerance test
Zhao et al., 2022 [51]	To investigate the effect of 8 wk TRE on adipose tissue transcriptome and glucose metabolism in confined obese men.	Low risk	One-group design, 8 wk	Duration of eating window	—	15	Obese men aged (mean ± SE) 63 ± 4 y	10 h TRE: - reduced BW, BMI, WC, BF, VF, and %BF	10 h TRE: - reduced fasting glucose and HbA1c - reduced breakfast glucose AUC using CGM - increased dinner venous blood glucose AUC
Kirkham et al., 2023 [52]	To evaluate the telephone-based delivery of weekday-only, ad libitum time-restricted eating on metabolic outcomes and concurrent lifestyle changes.	Low risk	One-group design, 8 wk	Duration of eating window	Ad libitum 8 h TRE (12:00–20:00) on weekdays	22	Breast cancer survivor women aged (mean ± SD) 66 ± 5 y	8 h TRE on weekdays: - reduced BW by 1 kg - no difference in BF, WC, BMI	8 h TRE on weekdays: - increased fasting insulin by 1.2 mIU/L - no difference in fasting glucose, HbA1c
Parr et al., 2023 [53]	To assess the effect of 5 d/wk, 9-h ad libitum TRE on 24-h glycemic control in adults with T2DM.	Low risk	One-group design, 4 wk	Duration of eating window	Habitual eating for 2 wk followed by 9 h TRE 5 d/wk (10:00–19.00) for 4 wk	19	T2DM men (*n* = 9) and women (*n* = 10) aged (mean ± SD) 50 ± 9 y	–	TRE vs. Habitual: - reduced 24 h AUC and MAD glucose, and postprandial (1 and 2h) glucose AUC - no difference in fasting glucose
Jones et al., 2020 [29]	To investigate the chronic effects of early TRE on the whole body and skeletal muscle insulin and anabolic sensitivity.	Low risk	Nonrandomized, parallel-design, 2 wk	Duration of eating window	TRE: ad libitum TRE at 08:00–16:00 (*n* = 8); or CON: control/caloric restriction diet for (*n* = 8)	16	Men aged (mean ± SEM) 23 ± 1 y	8h TRE vs. CON: - no difference in BW and BF	8h TRE vs. CON: - no difference in 24 h mean glucose - increased 24 h glucose CV by 5.1%
Lowe et al., 2020 [22]	To evaluate the effect of TRE on weight loss and metabolic risk markers.	Low risk	Randomized parallel-design, 12 wk	Duration of eating window	TRE: ad libitum TRE at 12:00–20:00 (*n* = 59); or CON: 3 structured meals per day (*n* = 57)	116	Men (*n* = 70) and women (*n* = 46) aged 18–64 y with BMI of 27–43 kg/m^2^	8 h TRE vs. CON: - no difference in BW, BF, VF, and SF	8 h TRE vs. CON: - no difference in fasting glucose, insulin, and HbA1c - no difference in HOMA-IR
Chow et al., 2020 [54]	To elucidate the effect of TRE with ad libitum intake on weight loss, BF reduction, and glucose metabolic parameter improvement.	Some concerns	Randomized parallel-design, 12 wk	Duration of eating window	TRE: self-defined TRE, generally at 10:40–18:40 (*n* = 11); or CON: ad libitum diet (*n* = 9)	20	Overweight men (*n* = 3) and women (*n* = 17) aged (mean ± SD) 45.5 ± 12.1 y	8 h TRE vs. CON: - reduced BW by 2.1 kg and VF by 0.3 kg - no difference in BF and %BF	8h TRE vs. CON: - no difference in fasting glucose, fasting insulin, HbA1c, mean glucose, CV, and SD - no difference in HOMA-IR and Matsuda Index
Moro et al., 2021 [55]	To evaluate the long-term effects of TRE on muscle mass and strength, fat mass, inflammation, and cardiovascular disease risk factors in healthy trained males.	Low risk	Randomized parallel-design, 12 mo	Duration of eating window	TRE: TRE at 13.00–20.00 (*n* = 11); or CON: eating window of 12 h at 8:00–20.00 (*n* = 9)	20	Men aged (mean ± SD) 29.21 ± 3.8 y	8 h TRE vs. CON: - reduced BW - no difference in BF and VF	—
Steger et al., 2023 [56]	To assess the effects of TRE on diet quality, appetite, and several eating behaviors.	Some concerns	Randomized parallel-design, 14 wk	Duration of eating window	TRE: early 8 h TRE (7:00–15:00) + energy restriction (*n* = 29); CON: ≥12 h eating schedule + energy restriction (*n* = 30)	59	Obese men (*n* = 12) and women (*n* = 47) aged (mean ± SD) 44 ± 11 y	TRE vs CON: - increased weight loss by 2.3 kg	—
Zaman et al., 2023 [57]	To compare 24 h glucose profiles and insulin sensitivity in participants after completing 12 wk of a behavioral weight loss intervention based on early TRE plus daily caloric restriction or daily caloric restriction alone.	Some concerns	Randomized parallel-design, 12 wk	Duration of eating window	TRE: TRE starting within 3 h of waking + daily caloric restriction (*n* = 23); CON: daily caloric restriction alone (*n* = 21)	44	men (*n* = 5) and women (*n* = 39) aged 18–50 with BMI 27–45 kg/m^2^	—	TRE vs. baseline (adjusted for BW): - no difference in glucose mean, SD, and CV; MAGE, fasting glucose, and insulin; HOMA-IR and HbA1c CON vs. baseline (adjusted for BW): - reduced SD by 1.8, CV by 0.01, MAGE by 5.4, insulin by 1.9, HOMA-IR by 0.4 Significant group × time interaction for MAGE
Martens et al., 2020 [58]	To evaluate the safety, tolerability, and feasibility of short-term TRE without weight loss in nonobese adults.	Low risk	Randomized crossover trial, 6 wk	Duration of eating window	TRE: TRE at 10:00–18:00 with dietary intake as indicated during the lead-in period; or CON: dietary intake similar to that during the lead-in period	22	Men (*n* = 10) and women (*n* = 12) aged 55–79 y with BMI of 24.7 ± 0.6 kg/m^2^	8 h TRE vs. CON: - no difference in BW	8 h TRE vs. CON: - no difference in fasting glucose and glucose AUC during OGTT
Correia et al., 2021 [36]	To evaluate the effects of long-term TRE without energy restriction on Wingate anaerobic test performance of well-trained, physically active healthy men.	Some concerns	Randomized crossover trial, 4 wk	Duration of eating window	TRE: ad libitum TRE at 13:00–21:00; or CON: ad libitum diet	12	Men aged (mean ± SD) 22.4 ± 2.8y	8h TRE vs. CON: - no difference in BW, BF, and %BF	—

Abbreviations: AUC, area under the curve; BMI, body mass index; BF, body fat; BW, body weight; CGM, continuous glucose monitoring; CI, confidence interval; CON, control; CV, coefficient of variation; HbA1c, glycated hemoglobin; HOMA-IR, homeostasis model assessment of insulin resistance; MAD, mean absolute deviation; MAGE, mean amplitude of glycemic excursion; OGTT, oral glucose tolerance test; RoB, risk of bias; SD, standard deviation; SE, standard error; SF, subcutaneous fat; T2DM, type 2 diabetes mellitus; TRE, time-restricted eating; VF, visceral fat; WC, waist circumference.

Six nonrandomized trials, including both one-group and parallel-design studies [29,37,[49], [50], [51], [52]], reported inconsistent findings regarding the impact of TRE on adiposity. Among these trials, 4 of them (*n* = 8‒22) reported weight reductions ranging from 1 to 3 kg after 8 to 16 wk of TRE among overweight participants [37,[49], [50], [51],^52^]. Moreover, 3 of these studies also observed reductions in BF percentage and WC [49,51]. However, in one nonrandomized trial (*n* = 16) involving borderline overweight participants (BMI of [mean ± SEM] 24.0 ± 0.6 kg/m^2^), no significant between-group differences in adiposity were observed [29]. Similarly, another nonrandomized trial (n=19) involving individuals with T2DM did not show significant changes in BW or BF after a 4-wk TRE intervention [50].

The 5 randomized trials included participants with diverse characteristics, including overweight or obese individuals, nonobese individuals, and physically trained individuals. Among the 3 randomized trials involving overweight or obese participants [22,54,56], 2 studies (*n* = 20‒59), rated as having “some concern” in the risk of bias analysis, found a significant reduction in mean BW following TRE by over 2 kg within 2 wk [54,56]. The third trial (*n* = 116) showed no effect on adiposity [22] but implemented a later TRE (eating only between 12:00–20:00) compared to the other 2 studies, which started TRE earlier at 07:00 and 10:40 [54,56]. Additionally, only 1 study (*n* = 59) observed a weight-loss effect when both TRE and control groups had similar energy intake [56], suggesting a potential independent effect of TRE on weight loss. In 1 randomized trial involving nonobese participants, TRE did not affect BW [58]. Among 2 trials involving physically trained participants who underwent TRE [36,55], only 1 study (*n* = 20) reported a decrease in BW after 12 mo of intervention (TRE: pre = 83.22 ± 5.92 kg, post = 80.33 ± 4.76 kg; control: pre = 84.64 ± 5.76 kg, post = 87.38 ± 4.39 kg; *P* < 0.05, values in mean ± SD) [55]. The discrepancy in findings between these 2 studies [36,55] might be attributed to the significantly shorter 4-wk follow-up period in the other trial (*n* = 12), in addition to the “some concern” rating in the risk of bias analysis [36].

Despite some compelling evidence suggesting that TRE may reduce adiposity, this effect has only been demonstrated in a limited number of studies with controlled energy intake. Further investigation is necessary to fully understand the role of TRE in influencing adiposity, considering factors such as timing, weight status, and the duration of follow-up.

#### Glucose homeostasis

Three observational studies investigating the relationship between the eating window and glucose homeostasis demonstrated inconsistent findings (Table 3). One study (*n* = 73) found no effect on HbA1c [45], although another (*n* = 118) unexpectedly demonstrated reduced insulin resistance, as indicated by lower HOMA-IR [47], despite an increase in 24 h mean, SD, and peak glucose concentration observed in the other study (*n* = 104) [48].

On the other hand, 4 randomized [22,54,57,58] and 4 nonrandomized trials [29,49,[51], [52]] involving healthy participants generally suggested a minimal impact of TRE on glucose parameters. For instance, with the exception of one study (*n* = 15) in obese men that reported a 0.3 mmol/L reduction in fasting glucose (baseline compared with week 8 [mean ± SD]: 5.7 ± 0.4 compared with 5.4 ± 0.4 mmol/L, *P* = 0.026) [51], fasting glucose and insulin concentration remained unchanged following TRE (*n* = 16‒116) [22,29,49,54,57,58]. CGM data in the first study showed no significant changes in 24-h glucose AUC, but glucose AUC did decrease in the morning and increased in the evening after standardized meals [51]. Another study (*n* = 16) found that 24-h glucose AUC was similar between the TRE and control groups in energy restriction, but participants on TRE experienced increased glycemic variability as indicated by a higher 24-h glucose CV (5.1%; 95% CI: 2.4, 7.8%, main effect (η^2^_p_) = 0.55) [29]. Also, conflicting results were observed regarding HbA1c, with again a reduction in the experimental study in which an effect on glucose was seen (baseline compared with week 8 [mean ± SD]: 6.1 ± 0.3 compared with 5.9 ± 0.3% unit, *P* = 0.008) [51] but not in the others [22,49,52,57]. Lastly, several trials found no significant differences in measures of insulin sensitivity such as HOMA-IR or Matsuda Index [22,49,54].

Two studies specifically involved individuals with T2DM. In one study (*n* = 19), participants on 9-h TRE experienced slightly lower glucose AUC after a test meal as compared to baseline values, nearly reaching statistical significance (TRE compared with control [mean ± SD]: 20 ± 4.6 compared with 21.4 ± 5.2 mmol·h/L, *P* = 0.06) [50]. The other study (*n* = 19) found that 8-h TRE lowered 24-h glucose AUC (TRE compared with habitual [mean ± SD]: −0.9 ± 1.4 mmol/L, *P* = 0.01), glucose mean absolute deviation (TRE compared with habitual [mean ± SD]: −0.2 ± 0.3 mmol/L, *P* = 0.01), and 2-h postprandial glucose AUC (−0.7 mmol/L·h, 95% CI: −0.3, −1.1, *P* = 0.001) [53]. In both mentioned studies [50,53], however, fasting glucose concentrations were unchanged.

Overall, implementing TRE in healthy participants has a negligible effect on various glucose parameters, according to the included articles in this category. Specifically, for T2DM participants, the results showed a lack of effect on glucose parameters, such as glucose AUC, with an effect observed in only 1 study. The null effect may have been due to insufficient statistical power. Moreover, the causality between TRE and glucose homeostasis is yet to be established due to the absence of randomized trials involving T2DM participants.

### Meal omission

#### Adiposity

A body of research from 6 cross-sectional studies [[59], [60], [61], [62], [63], [64]] suggests a potential link between skipping breakfast and increased adiposity (Table 4). A cross-sectional study (*n* = 499) observed that breakfast skippers were 4 times more likely to have a BMI >30 kg/m^2^ [59]. Similarly, other studies (*n* = 4218) demonstrated that skipping breakfast was significantly associated with increased risk of developing a BMI >25 kg/m^2^ in both men and women by >60%–120% [60,61] and in women alone by 40% [62]. Furthermore, a large-scale survey (*n* = 7007) in the United States revealed that individuals who consumed breakfast daily had a lower BMI (breakfast everyday compared with sometimes compared with rarely: 27.0 ± 0.3 compared with 27.9 ± 0.2 compared with 27.6 ± 0.3 kg/m^2^; *P* = 0.001) and a 5% lower prevalence of obesity compared to those who often skipped breakfast [63]. Additionally, a study conducted in Korea (*n* = 217) observed a 1 kg/m^2^ higher BMI among breakfast skippers who also reported irregular meal timing compared to regular eaters who eat breakfast (breakfast skippers and irregular meal timing compared with breakfast eater and regular meal timing: 24.4 ± 0.4 compared with 23.3 ± 0.4 kg/m^2^, *P* < 0.05) [64]. Despite these findings, 4 other studies (*n* = 32‒1401) did not demonstrate a significant association between skipping breakfast and adiposity measures such as BMI, BF percentage, or WC [67,68,[70], [71]]. Moreover, a lack of association with BMI was observed not only for the skipping of breakfast, but also of lunch and dinner in one article (*n* = 602) [65].

**TABLE 4:** Relationship of meal omission with adiposity and glucose homeostasis.

Authors	Primary aim(s)	RoB	Design, follow-up period	Definition of meal variation	Intervention/grouping	*n*	Participants	Findings on adiposity	Findings on glucose homeostasis
McCurley et al., 2022 [65]	To evaluate the association between hospital employees’ meal-skipping patterns and workplace food purchases, dietary quality, and cardiometabolic risk factors.	Good	Cross-sectional	Skipping meals	—	602	Men (n = 124) and women (n = 478) aged (mean ± SD) 43.6 ± 12. 2y	Breakfast, lunch, and dinner skippers vs. eaters: - no difference in BMI	Breakfast, lunch, and dinner skippers vs. eaters: - no difference in HbA1c
Kong et al., 2012 [66]	To investigate which self-monitoring behaviors, diet/eating-related weight loss strategies, and meal patterns were associated with weight change during dietary weight loss intervention among overweight-to-obese postmenopausal women.	Fair	Cohort, 12 mo	Skipping meals	Meal skippers; or Non-meal skippers	123	Overweight and obese postmenopausal women with mean age of 58 y	Meal skippers vs. Non-meal skippers (adjusted for covariates): - reduced weight loss by 4%	—
Ma et al., 2003 [59]	To investigate the association between eating patterns and obesity, controlling for physical activity and energy intake.	Good	Cross-sectional	Skipping breakfast	Breakfast eaters (*n* = 481); or Breakfast skippers (*n* = 18)	499	Men (*n* = 251) and women (*n* = 248) aged 20–70 y	Breakfast skippers vs. eaters (adjusted for covariates): - increased obesity risk by 350%	—
Song et al., 2005 [62]	To study the association between breakfast consumption and BMI in adults.	Fair	Cross-sectional	Skipping breakfast	Breakfast eaters (*n* = 3251); or Breakfast skippers (*n* = 967)	4218	Men (*n* = 2097) and women (*n* = 2121) aged ≥19 y	Breakfast skippers vs. eaters (adjusted for age and sex): - increased risk of overweight by 40% in women	—
Batista-Jorge et al., 2016 [60]	To investigate the association between breakfast omission and overweight or obesity.	Fair	Cross-sectional	Skipping breakfast	Breakfast eaters (*n* = 117); or Breakfast skippers (*n* = 223)	400	Men (*n* = 79) and women (*n* = 321) aged ≥18 y	Breakfast skippers vs. eaters (adjusted for covariates): - increased overweight and obesity risk by 120%	—
Widaman et al., 2016 [67]	To evaluate the association between chronic stress and variations in diet quality among breakfast eaters or breakfast skippers.	Fair	Cross-sectional	Skipping breakfast	Breakfast eaters (*n* = 40); or Breakfast skippers (*n* = 35)	75	Women aged 18–45 y	Breakfast skippers vs. eaters: - no difference in BW, BMI, and %BF	—
Forester et al., 2018 [68]	To compare insulin, leptin, glucagon-like peptide-1, ghrelin, peptide YY, and cholecystokinin response between habitual breakfast eaters and habitual skippers to a standard midday meal.	Fair	Cross-sectional	Skipping breakfast	Breakfast eaters (*n* = 18); or Breakfast skippers (*n* = 14)	32	Women aged (mean ± SD) 22.6 ± 3.3 y	Breakfast skippers vs. eaters: - no difference in BW, BMI, and BF	Breakfast skippers vs. eaters: - no difference in fasting glucose and insulin - no difference in HOMA-IR - no difference in glucose and insulin after test meal
Hashimoto et al., 2020 [69]	To evaluate the effects of skipping breakfast on glycemic parameters in T2DM patients.	Fair	Cross-sectional	Skipping breakfast	Breakfast eaters (*n* = 295); or Breakfast skippers (*n* = 22)	317	T2DM men (*n* = 180) and women (*n* = 137) aged (mean ± SD) 66.7 ± 10.7 y	—	Breakfast skippers vs. eaters (adjusted for covariates): - increased HbA1c by 0.5 % units
Pallangyo et al., 2020 [61]	To explore obesity prevalence and associated factors.	Fair	Cross-sectional	Skipping breakfast	Breakfast eaters; or Breakfast skippers	6691	Men (*n* = 3625) and women (*n* = 3066) aged 18–95 y	Breakfast skippers vs. eaters (adjusted for covariates): - increased overweight risk by 60%	—
Ding et al., 2020 [70]	To investigate the relationship between eating behaviors and hand grip strength in Chinese adults.	Fair	Cross-sectional	Skipping breakfast	Breakfast eaters (*n* = 1031); or Breakfast skippers (*n* = 370)	1,401	Men (*n* = 844) and women (*n* = 557) aged 20–55 y	Breakfast skippers vs. eaters: - no difference in BMI	—
Yoon et al., 2021 [64]	To assess the effect of regular mealtime and breakfast frequency on nutrient intake and cardiometabolic status in adult Koreans.	Good	Cross-sectional	Skipping breakfast	Breakfast ≥6×/wk and regular eating (HBRE) (*n* = 85); Breakfast ≥6×/wk and irregular eating (HBIE) (*n* = 20); Breakfast <6×/wk and regular eating (LBRE) (*n*=41); or Breakfast <6×/wk and irregular eating (LBIE) (*n*=71)	217	Men (*n* = 56) and women (*n* = 161) aged ≥19 y	Low frequency breakfast and irregular eaters vs. high frequency breakfast and regular eaters: - increased BMI by 1.1 kg/m^2^ and WC by 4.1 cm	LBIE vs. HBRE: - increased HOMA-IR by 1.55 - reduced HbA1c by 0.18%
Helo et al., 2021 [63]	To assess the association between skipping breakfast and all-cause and cancer-related mortality in the United States.	Good	Cross-sectional	Skipping breakfast	Breakfast every day (*n* = 4421); Some days (*n* = 1579); or Rarely (*n* = 1007)	7007	Men (*n* = 1,063) and women (*n* = 5,944) with mean age of 55.4 y	Breakfast everyday vs. some days: - reduced BMI by 0.9 kg/m^2^ and obesity prevalence by 5.1%	–
Saintila et al., 2023 [71]	To evaluate the association between the frequency of breakfast consumption and cardiometabolic risk in university teachers.	Fair	Cross-sectional	Breakfast frequency	Breakfast 0–2 d/wk (*n* = 27); Breakfast 3–5 d/wk (*n* = 43); or Breakfast 6–7 d/wk (*n* = 106)	176	Men (*n* = 64) and women (*n* = 112) aged (mean ± SD) 37 ± 8 y	Breakfast 3–5 d/wk vs. breakfast 6–7 d/wk (adjusted for covariates): - no difference in risk of BMI <25 and noncardiometabolic risk WC	Breakfast 3–5 d/wk vs. breakfast 6–7 d/wk (adjusted for covariates): - reduced risk of fasting glucose <110 mg/dL by 83%
Elahy et al., 2023 [72]	To assess the impact of hypothetical interventions on varying frequencies of breakfast and post-dinner snacks on weight maintenance after initial weight loss.	Good	Cohort, 12 mo	Skipping breakfast	Breakfast eaters (*n* = 245); or Breakfast skippers (*n* = 127)	372	Overweight and obese men (*n* = 108) and women (*n* = 264) aged 18–35 y	Breakfast skippers vs. eaters (adjusted for covariates): - increased BW regain by 1.39 kg - increased BF regain by 1 kg	—
Chowdhury et al., 2016 [73]	To evaluate the causal links between breakfast habits and energy balance components in free-living obese.	Some concerns	Randomized parallel-design, 6 wk	Skipping breakfast	Breakfast eaters (*n* = 11); or Breakfast skippers (*n* = 12)	23	Obese men (*n* = 8) and women (*n* = 15) aged 21–60 y	—	Breakfast skippers vs. eaters: - increased Δ fasting glucose by 0.3 mg/dL - no difference in fasting insulin - no difference in HOMA-IR and C-ISI Matsuda Index
Chowdhury et al., 2018 [74]	To investigate the effect of daily breakfast consumption or fasting until noon on acute metabolic and appetitive responses to a fixed breakfast and ad libitum lunch.	Low risk	Randomized parallel-design, 6 wk	Skipping breakfast	Breakfast eaters (*n* = 15); or Breakfast skippers (*n* = 16)	31	Lean men (*n* = 12) and women (*n* = 19) aged 22–56 y	—	Breakfast skippers vs. eaters: - no difference in 8-h plasma glucose and serum insulin
Parkner et al., 2011 [33]	To evaluate if skipping breakfast improves the daily glycemic profile in T2DM.	Some concerns	Randomized crossover trial, 2 d	Skipping breakfast	Breakfast eaters; or Breakfast skippers	13	T2DM patients aged 24–68 y	—	Breakfast skippers vs. eaters: - no difference in mean 19-h glucose - reduced 19-h glucose SD by 32%
Kobayashi et al., 2014 [34]	To evaluate the effect of breakfast skipping on diurnal variation of energy metabolism and blood glucose.	Some concerns	Randomized crossover trial, 24 h	Skipping breakfast	Breakfast eaters; or Breakfast skippers	8	Men aged (mean ± SEM) 25.3 ± 1.2 y	—	Breakfast skippers vs. eaters: - increased mean 24-h glucose by 6 mg/dL - increased mean glucose at 12:00–19:00 by 9 mg/dL and at 23:00–7:00 by 10 mg/dL
Ogata et al., 2019 [75]	To investigate the effect of 6 consecutive days of breakfast skipping and sedentary behavior on energy metabolism and glycemic control.	Low risk	Randomized crossover trial, 6 d	Skipping breakfast	Breakfast eaters; or Breakfast skippers	10	Men aged 20–30 y	—	Breakfast skippers vs. eaters: - increased mean 24-h glucose by 3.5 mg/dL in confinement on day 6 - no difference in mean 24-h glucose in free-living on day 1–5

Abbreviations: BF, body fat; BMI, body mass index; BW, body weight; C-ISI, composite insulin sensitivity index; HbA1c, glycated hemoglobin; HOMA-IR, homeostasis model assessment of insulin resistance; RoB, risk of bias; SD, standard deviation; SEM, standard error of the mean; T2DM, type 2 diabetes mellitus; WC, waist circumference.

In weight loss interventions, the influence of meal omission on BW was relatively consistent according to 2 cohort studies [66,74]. One study (*n* = 123) found that skipping any meal occasion was associated with significantly less weight loss compared to consuming all 3 meals (7.1%; 95% CI: 4.4, 9.8% compared with 11.4% ; 95% CI: 10.2, 12.6 of initial weight respectively, *P* = 0.005) [66]. Similarly, breakfast skippers, compared to breakfast eaters, experienced an average weight regain of 1.39 kg (95% CI: 1.45, 1.33) and BF regain of 1 kg (95% CI: 0.95, 1.06) 1 y after following a weight loss protocol (*n* = 372) [72].

These articles suggest that the evidence regarding the impact of meal omission, particularly breakfast skipping, on adiposity is rather inconsistent within the healthy population. It remains to be determined whether variations in outcomes are linked to differences in dietary patterns, metabolic profiles, and levels of physical activity across the general population. In contrast, despite being limited in number, cohort articles conducted in weight loss settings agreed that meal omission may interfere with weight loss.

#### Glucose homeostasis

Nine studies specifically investigated the association between meal omissions and glucose parameters in people without diabetes (Table 4) [34,64,65,68,71,[73], [74], [75]]. Among nondiabetics, several measures of glucose homeostasis, such as fasting glucose and insulin, did not differ between breakfast eaters and skippers in 2 studies (*n* = 31‒32) [68,74], as well as insulin sensitivity indexes (*n* = 23‒32) [68,73]. This trend was also observed in a study (*n* = 602) investigating the omission of any major meal occasion, with no difference in HbA1c between breakfast, lunch, or dinner skippers compared to eaters [65]. However, one study (*n* = 176) showed an improvement in glucose homeostasis (fasting glucose <110 mg/dL) with more frequent breakfast consumption of 6 to 7 d/wk [71]. Moreover, breakfast skippers had worse insulin sensitivity index (HOMA-IR 3.14 ± 0.6 compared with 1.59 ± 0.4) but lower HbA1c (5.34 ± 0.1 compared with 5.52 ± 0.1%) if they had irregular mealtimes compared to breakfast eaters with regular mealtimes (*n* = 217) [64], suggesting an interaction between breakfast omission and meal regularity. The positive association between breakfast omission and glucose parameters was also found in 3 randomized trials. One randomized trial (*n* = 23) showed that breakfast skipping caused elevated fasting blood glucose [73]. In a crossover trial (*n* = 10), nondiabetics following a breakfast skipping protocol in a confinement chamber showed higher mean 24-h glucose concentration (94.0 ± 7.5 compared with 90.5 ± 6.5 mg/dL, *P* = 0.003) with no difference in glycemic variability [75]. This result was reiterated by another crossover trial (*n* = 8) that showed breakfast skippers had a higher overall mean glucose level (89 ± 2 compared with 83 ± 3 mg/dL, *P* < 0.05), as well as at specific timeframes (12:00–19:00: 92 ± 2 compared with 83 ± 2 mg/dL, *P* < 0.05, 23:00–07:00: 83 ± 2 compared with 73 ± 3 mg/dL, *P* < 0.05) [34].

In participants with T2DM, conflicting associations between skipping breakfast and glucose parameters were observed in 2 studies [33,69]. In a cross-sectional study (*n* = 317), breakfast skippers showed a 0.5% unit higher HbA1c level [69]. In contrast, in a randomized crossover study, participants consuming breakfast showed higher glycemic variability (32%; 95% CI: 18, 49%; *P* < 0.0001) and a nonsignificant increase in mean glucose concentration (0.24 mmol/L; 95% CI: −0.02, 0.50 mmol/L; *P* = 0.066) [33]. The limited sample size (*n* = 13) in the latter article raises concerns about the statistical power, which may have contributed to the statistically nonsignificant findings.

Studies investigating both healthy and T2DM populations yielded mixed results on the effect of meal omission on glucose parameters. Notably, a randomized trial on T2DM participants observed an unexpected improvement in glucose variability with breakfast skipping, which warrants further investigation for confirmation. However, it may be important to note that the quality of more than half of the articles was rated as ‘fair” (observational) or as having ‘some concerns” (randomized trials), indicating limitations in their study design and potential biases. Consequently, the findings should be interpreted with caution.

### Meal timing

#### Adiposity

Three studies utilized the concept of caloric midpoint to characterize meal timing [47,76,77] (Table 5). The caloric midpoint refers to the time when an individual has consumed half of their TDEI. In one cross-sectional study (*n* = 718), university students with a caloric midpoint earlier than 15:00 had a lower BMI than those with a later caloric midpoint (23.0 ± 0.3 compared with 22.1 ± 0.2 kg/m^2^; *P* = 0.02) [77]. However, another cross-sectional study (*n* = 118) could not replicate this finding, demonstrating no association between caloric midpoint and several adiposity measures [47]. In a secondary analysis of a randomized controlled trial (RCT) (*n* = 188), participants with earlier caloric midpoints exhibited greater weight loss compared to those with later midpoints [76], supporting the notion that early eating patterns may lead to lower adiposity.

**TABLE 5:** Relationship of meal timing with adiposity and glucose homeostasis.

Authors	Primary aim(s)	RoB	Design, follow-up period	Definition of meal variation	Intervention/grouping	*n*	Participants	Findings on adiposity	Findings on glucose homeostasis
Garaulet et al., 2013 [21]	To investigate the role of food timing in weight-loss effectiveness in individuals who followed weight-loss treatment.	Good	Cross-sectional	Timing of lunch consumption	Early eaters: lunch before 15:00 (*n* = 199); or Late eaters: lunch after 15:00 (*n* = 212)	411	Men (*n* = 205) and women (*n* = 206) aged (mean ± SD) 42 ± 11 y undergoing weight loss therapy	Early vs. late eaters: - no difference in BMI, %BF, and WC	Early vs. late eaters: - reduced HOMA-IR by 0.4 points - no difference in fasting glucose and insulin
Leech et al., 2017 [78]	To investigate the relationship between temporal eating patterns, nutrient intakes, diet quality, and measures of adiposity in Australian adults.	Fair	Cross-sectional	Eating pattern derived from LCA according to lunchtime	Early eaters: lunchtime at conventional Australian mealtime (12:00) (*n* = 1972); or Late eaters: lunchtime at 1 h later (*n* = 1530)	4544	Men (*n* = 2127) and women (*n* = 2417) aged ≥19 y	Early vs. late eaters (adjusted for covariates): - no difference in BMI and WC - no difference in general and central obesity risk	—
Teixeira et al., 2019 [77]	To study the relationship of caloric midpoint with anthropometric measurements and macronutrient intake.	Fair	Cross-sectional	Caloric midpoint	Early eaters: caloric midpoint ≤15:00 (*n* = 383); or Late eaters: caloric midpoint >15:00 (*n* = 335)	718	Men (*n* = 233) and women (*n* = 485) aged (mean ± SD) 20.5 ± 2.9 y	Early vs. late eaters (adjusted for covariates): - reduced BMI by 1 kg/m^2^ - no difference in WC	—
Zhao et al., 2022 [45]	To investigate the relationship between eating architecture and body fat and markers of glycemic control in adults at increased risk of T2DM.	Good	Cross-sectional	The timing of the first and last meal	—	73	Men (n = 39) and women (n = 34) aged (mean ± SD) 58.8 ± 8.1 y	First or last meal timing (adjusted for covariates): - no association with BF	First or last meal timing: - no association with HbA1c
Dote-Montero et al., 2023 [47]	To investigate the association of meal timing with body composition and cardiometabolic risk factors in young adults.	Fair	Cross-sectional	Caloric midpoint, time from mid-sleep point to first food intake, and time from last food intake to mid-sleep point	—	118	Men (*n* = 36) and women (*n* = 82) aged (mean ± SD) 22 ± 2 y	Time from mid-sleep to first meal (adjusted for covariates): - no association with BMI, BF, and WC	Time from mid-sleep to first meal (adjusted for covariates): - associated with HOMA-IR (β = 0.294)
Azuma et al., 2023 [79]	To evaluate the relationship of breakfast eating habits with lifestyle behaviors and BMI although investigating sex-related differences.	Fair	Cross-sectional	Fasting duration after wake-up	—	254	Men (*n* = 76) and women (*n* = 178) aged (mean ± SD) 42 ± 12 and 36 ± 12y	Fasting duration after waking up correlated with: - correlated with BMI (among men skipping breakfast), *r* = 0.39 - inversely correlated with BMI (among women eating breakfast regularly), *r* = −0.21	—
Yoshimura et al., 2023 [48]	To investigate if an individual’s day-to-day nutrition-related lifestyle behaviors (meal timing, eating window, food intake, movement behaviors, sleep conditions, and BW) impact daily glycemic outcomes under free-living conditions.	Fair	Cross-sectional	Timing of breakfast, lunch, and dinner	—	104	Men (*n* = 44) and women (*n* = 60) aged (mean ± SD) 40 ± 12 y	—	Breakfast time: - inversely associated with 24-h glucose (β = −19.93), dinner time: - associated with 24-h glucose (β = 12.83)
Ma et al., 2003 [59]	To investigate the association between eating patterns and obesity, controlling for physical activity and energy intake.	Good	Cross-sectional	Interval between time out of bed and first eating; and interval between time of last episode of eating and time in bed	—	499	Men (*n* = 251) and women (*n* = 248) aged 20–70 y	The interval between the time out of bed and first eating: - no difference in obesity risk The interval between the time of last meal to bed time: - no difference in obesity risk	—
Garaulet et al., 2016 [80]	To study whether PLIN1, a circadian lipid-stabilizing protein in the adipocyte, interacts with the timing of food intake to affect weight loss.	Good	Cohort, 28 wk	Timing of lunch consumption	Early eaters: lunch before 15:00 (*n* = 96 TT+AT and 89 AA); or Late eaters: lunch after 15:00 (*n* = 122 TT+AT and 84 AA)	319	Overweight and obese men and women	Early vs. late lunch eaters (adjusted for covariates): - reduced BW by 3.5 kg in *PLIN1* AA carrier	—
Fleischer et al., 2022 [76]	To evaluate the association between aspects of food intake timing and higher weight loss.	Good	Cohort, 24 mo	Caloric midpoint and first and last meal timing	—	188	Men (*n* = 56) and women (*n* = 132) aged 20.7–50.8 y	1 h shift on first and last daily meal timing (adjusted for covariates): - reduced weight-loss by 1.4 and 0.6 kg	—
Vujovic et al., 2022 [81]	To determine the effects of late meal timing on mechanisms involved in energy intake control, energy expenditure, and molecular regulation of adipose tissue metabolism.	Some concerns	Randomized crossover trial, 6 d	Timing of eating window	Early eater: 8 h early eating window (08:00–16:00) Later eater: 8 h late eating window (12:00–20:00)	16	Women (*n* = 5) and men (*n* = 11) aged (mean ± SD) 37.3 ± 2.8 y	Early eater vs. later eater: - no difference in BW	—

Abbreviations: BF, body fat; BMI, body mass index; BW, body weight; HbA1c, glycated hemoglobin; HOMA-IR, homeostasis model assessment of insulin resistance; LCA, latent class analysis; PLIN1, Perilipin 1; RoB, risk of bias; SD, standard deviation; T2DM, type 2 diabetes mellitus; WC, waist circumference.

Meanwhile, 3 other studies evaluated lunch timing and its association with adiposity measures. Although lunch timing may not perfectly coincide with the caloric midpoint, it can influence the distribution of daily energy intake toward an earlier or later eating pattern. A cross-sectional study conducted in Australia (*n* = 4544) found no difference in BMI, WC, or risk of obesity between lunch at a conventional mealtime (12:00) and that consumed 1 h later [78]. Similarly, another cross-sectional study (*n* = 411) using a 15:00 cutoff time observed no difference in adiposity between early and late lunch eaters [21]. In contrast, a study (*n* = 319) investigating the effects of a weight loss intervention on individuals with a variant of *PLIN1* (rs1052700), a circadian lipid-stabilizing protein in adipocytes, revealed that AA carriers who ate lunch early lost an additional 3.5 kg compared to those who ate later (early compared with late lunch eaters in AA carriers [mean ± SE]: 10.63 ± 0.56 compared with late lunch eaters: 7.21 ± 0.67 kg, *P* < 0.001) [80]. This finding may suggest that mealtime-gene interactions play a significant role in adiposity.

Furthermore, although 3 cross-sectional studies (*n* = 73‒499) [45,47,61] found no association between the timing of the first or last meal and adiposity measures, a 2-y weight loss cohort study (*n* = 188) demonstrated that participants who advanced their first and last meals by 1 h experienced a 1.4% (95% CI: 0.785, 2.072%; *P* < 0.001) and 0.6% (95% CI: 0.031, 1.172%; *P* = 0.039) greater weight loss, respectively [76]. Although the study controlled for energy intake in its statistical models, it only provided instructions on physical activity restriction; hence, residual confounding by changes in physical activity may have affected these findings.

The absence of RCTs investigating the timing of caloric midpoint or lunch raises concerns about the reliability of the associations observed in observational articles. Without confirmatory evidence from RCTs, there is a risk of unknown confounders influencing the findings. This concern is further amplified by the findings of a single RCT (*n* = 16) that found no significant difference in BW between early and late eaters based on their eating windows [81]. Moreover, it is important to note that half of the observational articles in this category were rated ‘fair,” whereas the randomized trial received a ‘some concern” rating, highlighting potential limitations in methodological rigor.

#### Glucose homeostasis

The relationship between meal timing and glucose parameters remains inconclusive and complex according to 4 included articles (*n* = 73‒411) (Table 5) [21,45,47,48]. Eating lunch earlier in the day was associated with lower HbA1c [21], although the timing of the first or last meal did not appear to affect HbA1c [45]. Conversely, earlier breakfast (β: −19.93 mg/dL; 95% CI: −32.16, −7.71; *P* = 0.001) and later dinner times (β: 12.83 mg/dL; 95% CI: 4.31, 2.34; *P* = 0.003) were positively correlated to higher 24-h glucose concentration [48]. In contrast, another article [47] indicated that later breakfast deteriorates glucose homeostasis by increasing insulin resistance. This study showed that a longer interval between mid-sleep and consuming the first meal was associated with higher insulin resistance, as indicated by higher HOMA-IR scores (β: 0.294; *P* < 0.05). Meanwhile, the duration from the last meal to mid-sleep appeared not to affect insulin resistance. Finally, there was no difference in fasting glucose or insulin levels between individuals who eat lunch early or late [21].

Thus, the direction of association regarding the timing of the first, middle, and last meal on glucose homeostasis remains unclear due to conflicting results across the included articles. For instance, later breakfast may either improve or worsen glucose homeostasis, as measured by various glucose parameters.

### Within-day variation in dietary intake quantity

#### Adiposity

Four articles [19,[82], [83], [84]] identified an inverse association between the variation in the amount of dietary intake in the morning and adiposity measures, including BMI, BF, and WC, as well as reduced risk of overweight or obesity (Table 6). A cross-sectional study (*n* = 52) among men found that a higher intake of energy, protein, fat, and carbohydrates at breakfast and mid-morning snacks was inversely associated with BMI (*r* = −0.6), BF (*r* = −0.57), and WC (*r* = −0.6) [19]. A study (*n* = 872) that categorized participants by the percentage of their daily energy intake consumed in the morning found that those in the highest quintile (Q5) had >40% reduced risk of overweight or obesity compared with those in the lowest quintile (Q1) (adjusted OR: 0.53; 95% CI: 0.31, 0.89; *P* < 0.05) [82]. Similarly, a study that included a larger number of participants (*n* = 4243) showed that those in the Q4 of daily energy intake consumed at breakfast had 0.7 kg/m^2^ lower BMI compared with those in Q1 (Q4 compared with Q1: 27.4 compared with 26.7 kg/m^2^, *P* < 0.001) [83]. These findings were further supported by a study (*n* = 122) on weight loss that demonstrated that participants in the high-weight loss group (lost 6.1 ± 2.1 kg) tended to consume more energy ≤08:59 in the morning (high compared with low weight loss group: 15.0 ± 6.6 compared with 12.5 ± 5.8% TDEI, *P* = 0.03) as compared to the low weight loss group (loss 1.3 ± 2.3 kg) [84].

**TABLE 6:** Relationship of within-day variation in dietary intake quantity with adiposity and glucose homeostasis.

Authors	Primary aim(s)	RoB	Design, follow-up period	Definition of meal variation	Intervention/grouping	*n*	Participants	Findings on adiposity	Findings on glucose homeostasis
Dattilo et al., 2011 [19]	To study the association between energy distribution and macronutrient intake with body composition in healthy men and women.	Fair	Cross-sectional	Variation in the quantity of energy and nutrient intake consumed at each mealtime during the day	—	52	Men (*n* = 24) and women (*n* = 28) aged 20–45 y	BMI, %BF, and WC in men: - inversely correlated with morning energy, carbohydrate, protein, and fat intake - corelated with night fat intake %BF in women: - correlated with afternoon protein intake	—
Meule et al., 2014 [85]	To evaluate the association between night eating severity, BMI, and age in adults.	Fair	Cross-sectional	The difference in energy and nutrient intake at night	—	2317	Men (*n* = 1072) and women (*n* = 1245) aged ≥20 y	1-point lower of night eating severity score in the age groups of 31–70 y: - reduced BMI by 0.2 kg/m^2^	—
Hermenegildo et al., 2016 [83]	To evaluate the relation of energy intake distribution throughout the day on weight gain in adults.	Fair	Cross-sectional	The proportion of daily energy intake at each mealtime	—	4243	Men (*n* = 2117) and women (*n* = 2126) aged ≥18 y	Q4 vs. Q1 of % energy intake at breakfast (adjusted for age): - reduced BMI by 0.7 kg/m^2^ Q4 vs. Q1 of % energy intake at lunch (adjusted for age): - increased BMI by 0.7 kg/m^2^	—
McHill et al., 2017 [86]	To evaluate the association between the timing of food consumption relative to clock hour and endogenous circadian time, the composition of food intake, and body composition.	Fair	Cross-sectional	The proportion of daily energy intake consumed between 4 h before DLMO and sleep onset	—	110	Men (*n* = 64) and women (*n* = 46) aged 18–22 y	Percent energy intake consumed between 4h before DLMO and sleep onset: - correlated with %BF, *r* = 0.26	—
Xiao et al., 2019 [82]	To evaluate the associations between obesity and the timing of individual macronutrient intakes.	Good	Cross-sectional	The proportion of daily energy intake consumed at each mealtime	—	872	Men (*n* = 429) and women (*n* = 443) aged (mean ± SD) 63.5 ± 5.7 y	Q5 vs. Q1 of percent of TDEI in the morning (adjusted for age, sex, and race): - reduced overweight or obesity risk by 40% Q4 vs. Q1 percent of TDEI at night (adjusted for age, sex, and race): - increased overweight or obesity risk by 71%	—
Hunt et al., 2020 [87]	To assess meal frequency and consumption of low-calorie sweeteners in adults participated in the NHANES 2007–2016.	Fair	Cross-sectional	The ratio of meals consumed in the morning and evening	—	25,411	Men (*n* = 12,733) and women (*n* = 2678) aged ≥19 y	Q3 vs. Q1 of evening : morning energy ratio (adjusted for covariates): - reduced BMI by 0.36 kg/m^2^	—
Quist et al., 2021 [88]	To evaluate the association between late evening or night eating frequency and BMI and HbA1c among T2DM individuals.	Fair	Cross-sectional	The difference in energy and nutrient intake at night	Early eaters: after dinner and/or nighttime eating ≤2×/wk; or Late eaters: after dinner and/or nighttime eating ≥3×/wk	348	T2DM men and women aged (mean ± SD) 64.7 ± 10.8y	Late vs. early eaters: - no difference in BMI	Late vs. early eaters: - no difference in HbA1c
Jacob et al., 2020 [84]	To investigate the characteristics of low weight-loss responders by assessing the pre-diet distribution of daily energy and macronutrient intakes.	Good	Cohort, 12–15 wk	The difference in the cumulative percentage of energy and nutrient intake up to a certain time point	—	122	Men (*n* = 40) and women (*n* = 82) aged 39.1 ± 8.2 y in 12–15 wk energy restriction (−500 to −700 kcal/d)	High vs. low weight loss group: - increased cumulative energy and protein intake ≤08:59 - increased cumulative carbohydrate intake ≤08:15, 14:29, and 16:59	—
Elahy et al., 2023 [72]	To assess the impact of hypothetical interventions on varying frequencies of breakfast and post-dinner snacks on weight maintenance after initial weight loss.	Good	Cohort, 12 mo	Post-dinner snacks consumption	Post-dinner snacks 0–2 times/wk; or Post-dinner snacks 3–7 times/wk	372	Overweight and obese men (*n* = 108) and women (*n* = 264) aged 18–35 y	Post-dinner snacks 0–2 times/wk vs. 3–7 times/wk - reduced BW regain by 1.18 kg - reduced BF regain by 1.23 kg	—
Oliveira et al., 2023 [89]	To evaluate whether recommending regular consumption of a low-carbohydrate breakfast instead of a low-fat breakfast could result in significant enhancements in glycemic control for individuals with T2DM.	Low risk	Randomized parallel-design study, 12 wk	The proportion of daily carbohydrate and fat intake consumed at breakfast	LC: low-carbohydrate breakfast (∼465 kcal, P:F:C = 22:72:6% energy; or CON: low-fat breakfast (∼450 kcal, P:F:C = 18:31:51% energy	121	T2DM men (*n* = 57) and women (*n* = 64) aged (mean ± SD) 64 ± 9 y	LC vs. CON: - no difference in BW, BMI, WC	LC vs. CON: - reduced mean glucose by -0.7 mmol/L, SD glucose by −0.2 mmol/L and MAGE by −0.8 - no difference in HbA1c
Hibi et al., 2013 [35]	To assess the effects of a 2 wk nighttime vs. daytime snacking intervention on lean young women’s energy, glucose, and lipid metabolism.	Some concerns	Randomized crossover trial, 13 d	Timing of snack consumption	Daytime snack (192.4 ± 18.3 kcal) at 10:00; or Nighttime snack (192.4 ± 18.3 kcal) at 23:00	11	Women aged (mean ± SD) 23 ± 1 y	—	Daytime vs. nighttime snacking: - no difference in fasting glucose & insulin - no difference in AUC glucose & insulin after OGTT
Kessler et al., 2017 [90]	To investigate the effects of a diet with fat mainly eaten in the morning and carbohydrates in the evening, and vice versa, on whole-day levels of glucose, glucose-regulating hormones, and glucose tolerance.	Low risk	Randomized crossover trial, 4 wk	The difference in the proportion of daily carbohydrate and fat intake in the morning and the evening	Carbohydrate-rich meals until 13:30 (P:F:C=15:20:65% energy) and fat-rich meals at 16:30–22:00 (P:F:C=15:50:35% energy); or Fat-rich meals until 13:30 (P:F:C=15:50:35% energy) and carbohydrate-rich meals at 16:30–22:00 (P:F:C=15:20:65% energy)	29	Nonobese men (*n* = 29) with both normal (*n* = 18) or impaired fasting glucose or glucose tolerance (*n* = 11)	Morning carbohydrate vs. fat-rich meal: - no difference in BW and BMI	Morning carbohydrate vs. fat-rich meal: - no difference in fasting glucose, insulin, HOMA-IR
Kuwahara et al., 2022 [91]	To assess the effect of different nutrient balances during lunch on glucose level variability.	Some concerns	Randomized crossover trial, 4 d	The proportion of daily macronutrient intake consumed at lunch	Standard lunch (P:F:C=18:36:46% energy); Protein-rich lunch (P:F:C=57:27:16% energy); Fat-rich lunch (P:F:C=17:68:15% energy); or Carbohydrate-rich lunch (P:F:C=12:13:75% energy)	14	Men (*n* = 8) aged (mean ± SD) 24.6 ± 5.6 y and women (*n* = 6) aged (mean ± SD) 23.8 ± 4.6 y	—	Fat-rich lunch vs. standard lunch: - increased iAUC 2 h after dinner by 61%; Fat-rich lunch vs. protein-rich lunch: - increased iAUC 2 h after dinner by 87%

Abbreviations: BF, body fat; BMI, body mass index; BW, body weight; CON, control; DLMO, dim-light melatonin onset; HbA1c, glycated hemoglobin; HOMA-IR, homeostasis model assessment of insulin resistance; (i)AUC, (incremental) area under the curve; LC, low carbohydrate; MAGE, mean amplitude of glucose excursion; OGTT, oral glucose tolerance test; P:F:C , protein: fat: carbohydrate ratio; RoB, risk of bias; T2DM, type 2 diabetes mellitus; TDEI, total daily energy intake; WC, waist circumference.

Conversely to the beneficial effects observed of meal intake in the morning, 4 observational studies [19,72,85,86] linked increased dietary intake during nighttime with higher adiposity measures. For example, a high night-eating severity score was associated with a 0.2 kg/m^2^ higher BMI (*n* = 2317) [85]. Additionally, evening meal and supper consumption were positively correlated with BMI, BF, and WC in men (*n* = 52) [19]. This finding was further supported by a study (*n* = 110) that observed a positive correlation (*r* = 0.26) between dietary intake near sleep time, defined as the period between 4 h before the time of dim-light melatonin onset and sleep onset, with BF [86]. One cohort study (*n* = 372) with a 1-y follow-up demonstrated that avoiding post-dinner snack consumption led to a reduction in BW (−1.18 kg; 95% CI: −1.22, −1.14 kg) and BF regain (−1.23 kg; 95% CI: −1.27, −1.19) after weight loss treatment [72]. However, 2 studies [88,89] showed contradictory results. One of these studies (*n* = 25,411) found that individuals in the third quartile of the evening-to-morning energy ratio had a 0.36 kg/m^2^ lower BMI than those in the lowest quartile (−0.36 kg/m^2^; 95% CI: −0.68, −0.05 kg/m^2^) [87], indicating an improvement of adiposity with higher energy intake in the evening. Additionally, frequent dietary intake during nighttime (≥3×/wk) was not associated with increased BMI in diabetic participants (*n* = 348) [88].

Although observational articles have provided valuable insights into the relationship between meal timing and BW, the lack of RCTs in this research area limits their definitive conclusions. Two trials sought to address this gap by investigating the effects of meals with different macronutrient compositions at specific mealtimes rather than examining variations in energy intake alone. However, neither study found any significant differences in adiposity measures, including BW and BMI [89,90]. The limitations of article methodologies warrant cautious interpretation of the findings because only 3 of 9 observational studies in this category were rated as “good” [72,82,84], and half of the randomized trials were considered having low risk of bias [89,90].

#### Glucose homeostasis

Interventions that induced gaps in the quantity of energy or nutrient intake between morning and evening or nighttime yielded varied results on various glucose homeostasis parameters (Table 6). For example, providing snacks either during the day or at night did not impact fasting glucose, fasting insulin, or glucose and insulin AUC during an OGTT [35]. Moreover, an study involving participants with T2DM somewhat supported these results as HbA1c was unaltered with unchanged frequencies of nighttime eating [88].

In 3 other studies [[89], [90], [91]], investigators manipulated the macronutrient composition at a particular mealtime and observed the effect on glucose parameters. Low-carbohydrate, high-fat breakfast intake (low-carbohydrate compared with low-fat breakfast, protein: fat: carbohydrate (P:F:C) 22:72:6% compared with 18:31:51% energy in breakfast) decreased 24-h mean glucose concentration (−0.7 mmol/L; 95% CI: −1.4, −0.1 mmol/L; *P* = 0.03) and its variation according to differences in SD (−0.2 mmol/L; 95% CI: −0.4, −0.1 mmol/L; *P* = 0.009) and mean amplitude of glycemic excursion (MAGE) (−0.8; 95% CI: −1.2, −0.3; *P* = 0.002) in one study (*n* = 121) [89], but did not influence fasting glucose, insulin, or HOMA-IR in another (*n* = 29) [90]. Furthermore, increasing the fat proportion during lunch (P:F:C = 17:68:15% energy in lunch) significantly increased 2-h glucose iAUC at dinner time, by 61% and 87% compared to a standard lunch (P:F:C=18:36:46% energy in lunch) and protein-rich lunch (P:F:C=57:27:16% energy in lunch), respectively (standard lunch: 57.5 ± 37.4, protein-rich lunch: 49.4 ± 28.0, fat-rich lunch: 92.4 ± 41.4, values in mean ± SD) (*n* = 14) [91].

Our review highlights a lack of evidence regarding the effect of higher energy intake in the morning or at night on glucose control. Similarly, although the effects of prioritizing specific macronutrients at different mealtimes remain inconclusive and incomplete, promising findings warrant further exploration to identify potentially beneficial dietary strategies. These strategies should encompass optimizing macronutrient distribution at specific mealtimes to improve glucose homeostasis.

### Temporal distribution

#### Adiposity

Dietary data that describes the contribution of each mealtime to TDEI or the magnitude of within-day variation in dietary intake is categorized as temporal distribution (Table 7). Four articles [93,96,98,101] explicitly displaying different types of dietary intake temporal distribution did not demonstrate a difference in adiposity. For instance, in an observational study (*n* = 192), dietary patterns derived from hierarchical cluster analysis, identifying when peak energy intake occurred during meals, were not associated with BW, BMI, and WC [93]. Three randomized trials (*n* = 26‒51), rated as having a low risk of bias, were in line with those articles in that they found no effect on BW and several other adiposity measures with a skewed distribution of energy between evening and morning (breakfast: lunch: dinner/B:L:D = 54:35:11% compared with 11:35:54%) [96], (B:L:D = 45:35:20% compared with 20:35:45%) [101], or of protein intake (B:L:D = 26:36:38% compared with 9:32:60%) [98].

**TABLE 7:** Relationship of temporal distribution with adiposity and glucose homeostasis.

Authors	Primary aim(s)	RoB	Design, follow-up period	Definition of meal variation	Intervention/grouping	*n*	Participants	Findings on adiposity	Findings on glucose homeostasis
Farsijani et al., 2016 [92]	To assess the association of protein quantity and distribution with lean and appendicular lean mass and their 2-y decline in older adults.	Good	Cross-sectional	CV of within-day protein intake	—	712	Men (*n* = 351) and women (*n* = 361) aged 67–84 y	Q4 vs. Q1 protein intake CV in males and females: - no difference in BW, BMI, BF, WC	—
Horn et al., 2022 [93]	To evaluate the dietary intake pattern differences among obese individuals.	Fair	Cross-sectional	Meal pattern derived from hierarchical cluster analysis corresponding to the time of day with the highest energy intake peak.	—	192	Men (*n* = 91) and women (*n* = 101) aged 20–5 5y	Dinner- vs. lunch- vs. supper- vs. midnight- vs. regular-eaters: - no difference in BW, BMI, and WC	—
Jacob et al., 2023 [94]	To assess the associations between late eating and BMI.	Fair	Cross-sectional	%TDEI after specific time	—	301	Men (*n* = 133) and women (*n* = 168) aged (mean ± SD) 38.7 ± 8.5 y	%TDEI after 17:00 or 20:00 (adjusted for covariates): - not correlated with BMI	—
Farsijani et al., 2020 [95]	To evaluate the effect of within-day protein intake distribution on body composition improvements in overweight/obese older adults during a hypocaloric and exercise intervention.	Good	Cohort, 1 y	CV of within-day protein intake	—	36	Overweight and obese men (*n* = 6) and women (*n* = 30) aged (mean ± SD) 70.6 ± 6.1 y	1-point reduction of protein intake CV (adjusted for energy intake): - reduced BMI by 5.3 kg/m^2^ and subcutaneous fat by 161 cm^2^	—
Jakubowicz et al., 2013 [96]	To evaluate the effects of different meal timing distributions on insulin resistance and hyperandrogenism in lean PCOS patients.	Low risk	Randomized parallel-design study, 90 d	The proportion of daily energy intake consumed at each mealtime	High-energy breakfast: B:L:D=54:35:11% (*n* = 25); or Low energy breakfast: B:L:D=11:35:54% (*n* = 26)	51	Lean (BMI<24.9 kg/m^2^) women with PCOS aged (mean ± SD) 31.96 ± 3.82 y	High-energy breakfast vs. baseline: - no difference in BW, BMI, and WC	High-energy breakfast vs. baseline: - reduced AUC glucose by 20% and insulin by 49% after OGTT
Hudson et al., 2017 [97]	To assess the effects of within-day protein intake distribution on body composition during energy restriction and resistance training.	Low risk	Randomized parallel-design study, 16 wk	The proportion of daily protein intake consumed at each mealtime	Energy-restricted diet of 750 kcal/d with: Even protein distribution: B:L:D=33:33:33% (*n* = 21); or skewed protein distribution: B:L:D=11:22:66% (*n* = 20)	41	Men (*n* = 15) and women (*n* = 26) aged (mean ± SEM) 35 ± 2 y with BMI 31.5 ± 0.5 kg/m^2^	Even vs. skewed group: - no difference in BW, BMI, WC, HC, WHR, BF, and %BF	—
Kim et al., 2018 [20]	To evaluate the chronic effects of protein intake pattern at 1.1 g protein/kg/day in mixed meals on lean body mass, functional outcomes, whole body protein kinetics, and muscle protein fractional synthesis rate over 8 wk.	Some concerns	Randomized parallel-design study, 8 wk	The proportion of daily protein intake consumed at each mealtime	Even protein distribution: B:L:D=33:33:33% (*n* = 7); or skewed protein distribution: B:L:D=15:20:65% (*n* = 7)	14	Men (*n* = 6) and women (*n* = 8) aged 51–69 y	—	Even vs. skewed: - no difference in 23-h glucose and insulin AUC
Nouripour et al., 2021 [30]	To evaluate the effect of high protein or high carbohydrate intake at the evening meal on metabolic parameters of T2DM patients.	Low risk	Randomized parallel-design study, 10 wk	The proportion of daily carbohydrate and protein intake consumed at each mealtime	Standard meal: evenly distributed protein & carbohydrate (*n* = 36); High carbohydrate evening: 40%–45% carbohydrate at dinner (*n* = 31); or High protein evening: 40%–45% protein at dinner and evening snack (*n* = 29)	96	T2DM men (*n* = 46) and women (*n* = 60) aged 32–65 y	High protein evening vs. standard meal: - reduced weight-loss by 0.8 kg and BMI reduction by 0.28 kg/m^2^	High protein evening vs. standard meal: - Reduced HbA1c by 0.19%
Yasuda et al., 2020 [98]	To evaluate if a protein-enriched meal at breakfast is more effective for muscle accretion vs. the typical skewed protein intake pattern.	Low risk	Randomized parallel-design study, 12 wk	The proportion of daily protein intake at each mealtime	Resistance training 3×/wk and: High protein breakfast: B:L:D=26:36:38% (*n* = 12); or Low protein breakfast: B:L:D=9:32:60% (*n* = 12)	26	Men aged (mean ± SEM) 20.8 ± 0.4 y	High vs. low protein breakfast: - no difference in BW, BMI, and BF	—
Beebe et al., 1990 [31]	To investigate the effect of 3 temporal energy intake patterns on glucose, insulin, and C-peptide concentration.	Some concerns	Randomized crossover trial, 26 h	The proportion of daily energy intake consumed at each mealtime	Even energy distribution: B:L:D=30:40:30%; Even energy distribution with snack: B:L:D=20:20:30% and 10% snack after each meal; or skewed energy distribution: B:L:D=10:20:70%	6	Moderately controlled T2DM men (*n* = 4) and women (*n* = 2) aged 35–64 y	—	Even vs. skewed energy distribution: - increased mean increment of insulin secretion rate from fasting level 08:00—00:00 by 40% - no difference in mean glucose and insulin 08:00—00:00
Keim et al., 1997 [32]	To evaluate the effect of large morning meals vs. large evening meals on body weight, body composition, and energy utilization during weight loss.	Some concerns	Randomized crossover trial, 6 wk	The proportion of daily energy intake consumed in the morning and at night	Early eaters: 70% energy as 2 meals in the morning; or Late eaters: 70% energy as 2 meals in the evening	10	Women aged 23–39 y	Early vs. late eaters: - increased weight loss by 0.63 kg	—
Parr et al., 2018 [99]	To evaluate the effect of high vs. low energy intake on the first meal on glucose and insulin responses during prolonged sitting in individuals with prediabetes.	Low risk	Randomized crossover trial, 24 h	The proportion of daily energy intake consumed at each mealtime	High-energy breakfast: B:L:D=50:30:20%; or Low energy breakfast: B:L:D=20:30:50%	13	Overweight and obese and prediabetic men (*n* = 6) and women (*n* = 7) aged (mean ± SD) 60 ± 6 y	—	High vs. low energy breakfast: - increased glucose AUC between 08:00–18:00 by 4.43 mmol/L·h and 24-h insulin AUC by 474 mmol/mL·h - no difference in mean 24-h glucose - no difference in SD, MAGE, and CONGA
Singh et al., 2020 [100]	To investigate the circadian-restricted feeding on parameters of metabolic syndrome in healthy subjects.	Some concerns	Randomized crossover trial, 4 wk	The proportion of daily energy intake consumed in the morning and at night	Early eaters: most energy (2000 kcal) at 8:00–9:30; or Late eaters: most energy (2000 kcal) at 20:00–21:30	22	Men (n = 20) and women (n = 2) aged (mean ± SD) 30.09 ± 8.95 y	Early vs. late eaters: - reduced BW by 0.8 kg, BMI by 0.3 kg/m^2^	Early vs. late eaters: - reduced HbA1c by 0.28 % units
Ruddick-Collins et al., 2022 [101]	To test if the consumption of controlled diets with the largest meal of the day at breakfast compared to that in the evening produces higher weight loss.	Low risk	Randomized crossover trial, 4 wk	The proportion of daily energy intake consumed at each mealtime	High-energy breakfast: B:L:D=45:35:20%; or Low-energy breakfast: B:L:D=20:35:45%	30	Overweight or obese men (*n* = 16) and women (*n* = 14) aged (mean ± SD) 50.9 ± 2.1 y	High vs. low energy breakfast: - no difference in BW, BMI, %BF, WC, HC, WHR, and weight loss	High vs. low energy breakfast: - no difference in fasting glucose, fasting insulin & HbA1c - no difference in HOMA-IR

Abbreviations: AUC, area under the curve; B:L:D, breakfast: lunch: dinner; BF, body fat; BMI, body mass index; BW, body weight; CONGA, continuous overall net glycemic action; CV, coefficient of variation; HbA1c, glycated hemoglobin; HC, hip circumference; HOMA-IR, homeostasis model assessment of insulin resistance; MAGE, Mean amplitude of glucose excursion; OGTT, oral glucose tolerance test; PCOS, polycystic ovary syndrome; RoB, risk of bias; SD, standard deviation; SEM, standard error of the mean; T2DM, type 2 diabetes mellitus; TDEI, total daily energy intake; WC, waist circumference; WHR, waist-hip ratio.

Concentrating a large proportion of daily energy intake in a particular mealtime may be a critical factor in how the temporal distribution of dietary intake influences adiposity, as reported in 2 articles [32,100]. For instance, when 70% TDEI was allocated in the morning, participants (*n* = 10) undergoing weight loss treatment lost 0.63 kg more weight compared to those with 70% TDEI allocated in the evening (70% TDEI in the morning compared with evening [mean ± SEM]: −3.9 ± 0.19 compared with −3.27 ± 0.26 kg; *P* < 0.01) [32]. Similarly, consuming most energy (2000 kcal) at 08:00–09:30 compared to 20:00–21:30 induced a lower mean BW by 0.8 kg (95% CI: 0.53, 1.06) and BMI by 0.3 kg/m^2^ (95% CI: 0.20, 0.41) (*n* = 22) [100]. The interpretation of findings from both articles requires caution, however, as both received a ‘some concern” quality rating.

Three articles evaluated the association between protein intake temporal distribution and adiposity measures [20,92,97]. The within-day variation of protein intake, expressed by CV and indicating the degree of daily between meal protein intake variability, was not associated with BW, BMI, BF, and WC in an observational study (*n* = 712) [92]. This result was in line with 2 randomized trials (*n* = 14‒41) evaluating the effect of even compared with morning-skewed protein intake distribution in overweight young (B:L:D = 33:33:33% compared with 11:22:66% total daily protein intake) [97] and older adults (B:L:D = 33:33:33% compared with 15:20:65% total daily protein intake) [20] that found no difference in several adiposity measures.

On the other hand, the effect of protein intake distribution was particularly apparent in weight loss settings. The combination of physical activity, energy restriction, and lower protein intake CV was associated with lower BMI and subcutaneous fat area after 1 y (*n* = 36) [95]. In a trial (*n* = 96) employing energy restriction of 219 to 297 kcal/d from the baseline, T2DM participants, of whom half or more were overweight and who received 40% to 45% total daily protein intake in the evening, had less weight loss (even compared with skewed protein intake [mean ± SD]: −1.3 ± 1.2 kg compared with −2.1 ± 2.1 kg, *P* < 0.05) and less BMI reduction (even compared with skewed protein intake [mean ± SD]: −0.48 ± 0.47 compared with −0.76 ± 0.74 kg/m^2^, *P* < 0.05) after 10 wk as compared to participants who received an evenly distributed protein intake over the day [30]. These results suggest that under specific circumstances, such as following an energy-restricted diet, the way protein is distributed throughout the day could affect adiposity.

Research on the impact of temporal distribution of dietary intake, with a focus on energy and protein distribution, on adiposity revealed diverse results, likely due to varying study conditions. For instance, altering energy intake to be higher in the morning or evening, although maintaining substantially high intake during that timeframe, yielded an effect on adiposity. Similarly, articles examining protein distribution suggested that evenly spreading protein throughout the day only affects BW in weight loss scenarios, potentially indicating an interaction between energy restriction and protein intake distribution.

#### Glucose homeostasis

Studies investigating the association between temporal distribution of dietary intake and glucose homeostasis suggest that consuming a substantially high-energy breakfast leads to improvements in glucose metabolism, as indicated by favorable changes in glucose parameters (Table 7). In lean females (*n* = 51) who consumed a high-energy breakfast (B:L:D = 54:35:11% TDEI) for 90 d, postprandial AUC glucose was reduced by 20% (baseline [mean ± SE]: 17.43 ± 0.16; day 90: 13.92 ± 0.07 mg/dL·120 min, *P* < 0.0001) and insulin by 49% (baseline [mean ± SE]: 7.36 ± 16; day 90: 3.78 ± 94 μIU/mL·120 min, *P* < 0.0001) compared to baseline. In contrast, a low-energy breakfast (B:L:D = 11:35:54% TDEI) only resulted in a 7% (baseline: 7.80 ± 16; day 90: 7.19 ± 16 μIU/mL·120 min, *P* < 0.05) reduction in postprandial AUC insulin, but not in glucose, as compared to baseline. This effect of diet (high- compared with low-energy breakfast) was shown to be statistically significant [96]. Furthermore, extreme allocation of energy intake (2000 kcal) in the morning at 08:00–09:30 induced HbA1c reduction of 0.28% unit (95% CI: 0.23, 0.33% unit; *P* < 0.001) compared to the same amount consumed at 20:00–21:30 (*n* = 22) [100]. However, 2 other studies failed to demonstrate significant alterations in various glucose parameters, such as 24h mean glucose concentration, SD, MAGE, continuous overall net glycemic action (*n* = 13) [99], fasting glucose, fasting insulin and HbA1c, and HOMA-IR (*n* = 30) [101] when 45%–50% of energy intake was allocated at breakfast, which is a lower proportion than that in the previous articles.

Among individuals with T2DM (*n* = 6), morning-skewed energy intake (B:L:D = 10:20:70 compared with 30:40:30% TDEI) induced lower peak glucose, peak insulin, and insulin secretion rate in the morning but not in the afternoon and evening, despite no influence on 24-h mean glucose and insulin concentration [31]. Furthermore, although 24-h metrics derived from CGM appeared to show no change between dietary intake distributions, another trial (*n* = 96) indicated higher HbA1c reduction by 0.19% unit (even compared with skewed protein intake [mean ± SD]: −0.45 ± 0.36 compared with −0.26 ± 0.36% unit, *P* < 0.05) in T2DM participants with even protein intake distribution compared to those with 40%–45% protein intake in the evening [30], suggesting that evenly distributed protein intake may be favored in this case. However, a similar intervention in healthy participants (*n* = 14) did not show an effect on glucose parameters, specifically glucose and insulin AUC [20].

Included studies in the temporal distribution category revealed that only manipulating time-of-day energy intake with a significant portion allocated to morning or evening impacted glucose parameters, aligning with research on the link between temporal distribution of energy intake and adiposity. Interestingly, modifying protein intake distribution only affected glucose control in T2DM participants, not healthy individuals, suggesting limited generalizability if confirmed.

A simplified summary of the findings regarding the association on how different dietary intake variations affect adiposity and glucose homeostasis can be found in Table 8.

**TABLE 8:** A simplified overview of the number of studies and their findings on how different dietary intake variations affect adiposity and glucose homeostasis.

Dietary intake variations	Design	Adiposity			Glucose homeostasis		
		↓	↔	↑	↓	↔	↑
Between-day variation in dietary intake quantity	Obs	—	1	5	—	2	—
	Exp	—	2	—	—	1	—
Between-day variation in meal timing	Obs	—	1	4	—	2	—
	Exp	—	—	—	—	—	—
Eating windows	Obs	—	2	—	1	1	1
	Exp	—	5	7	1	7	2
Meal omission	Obs	—	5	8	—	3	2
	Exp	—	—	—	1	1	3
Meal timing	Obs	—	5	4	—	1	2, 1^1^
	Exp	—	1	—	—	—	—
Within-day variation in dietary intake quantity	Obs	—	1	8^2^	—	1	—
	Exp	—	2	—	—	2	2^3^
Temporal distribution	Obs	—	1^4^ + 2^5^	1^4^	—	—	—
	Exp	—	2^5^ + 2^6^	2^5^ + 1^6^	—	3^5^ + 1^6^	2^5^ + 1^6^

Numbers represent study count.

↓/↑/↔: associated with lower/higher/no association (or contradictory results within study) with adipose or glucose homeostasis parameter.

Abbreviations: Obs, observational studies; Exp, experimental studies.

1 From earlier breakfast and late dinner.

2 From the tendency to later eating.

3 From fat-rich meals in a mealtime.

4 From high protein intake variability.

5 From the tendency to have more energy consumed in later time/skewed toward morning energy intake distribution.

6 From the tendency to have more protein consumed in later time/skewed toward morning protein intake distribution.

## Discussion

In this systematic review, we hypothesized that aligning dietary intake with the circadian rhythm may positively impact metabolic health, as indicated by reduced adiposity and improved glucose homeostasis. We argue that for optimizing circadian-related health, dietary intake should adhere to appropriate meal timings and maintain consistency both in timing and amount of energy and nutrient intake. Motivated by the potential influence of these factors on adiposity and glucose regulation, we analyzed both between-day (regularity across days) and within-day (timing and distribution within a day) variations in dietary intake. Our focus on adiposity as the primary outcome purposely limits our review to energy and macronutrient intake, specifically protein, fat, and carbohydrates, due to their direct effect on energy metabolism. In this systematic review, the measures of glucose regulation were regarded as secondary outcomes, potentially improving as a result of reduced adiposity [102]. However, diet may also directly influence these measures [90]. Efforts were made to describe the available evidence coherently despite discrepancies in outcomes and the variations in exposures and study designs.

Findings in the literature were primarily limited by inconsistent results among studies and the absence of randomized trials. Thus, firm conclusions regarding causal effect could not be drawn in several categories of variation in dietary intake. Nevertheless, for several exposures and outcomes, randomized trials were available with consistent results. This was the case with studies on the effect of meal omission on glucose parameters in healthy participants, which was supported by 3 randomized trials that showed elevated fasting blood glucose or mean glucose concentration due to skipping breakfast [34,73,75]. However, we observed that 2 of the 3 randomized trials received a rating of “some concern.” Consequently, we advise readers to interpret these findings with appropriate caution. For the other exposures and outcomes, given the insufficient evidence, we examined potential factors that could account for the inconsistencies observed in the results. These included variations in participant characteristics or conditions during the experiments and the quantity of food or energy consumed during specific mealtimes.

Discrepancies observed across studies may be attributed to variations in participant characteristics. For instance, individuals with T2DM may be more responsive to dietary interventions, making them more likely to exhibit positive associations. Research has shown that morning-skewed protein distribution reduces various glucose parameters among T2DM patients but not in healthy individuals [20,30]. Additionally, factors like energy adequacy or deficiency may also influence the outcomes. In weight loss approaches incorporating energy restriction, a more evenly distributed protein intake was associated with greater weight loss [30,95]. However, the effect of this dietary intervention was not observed in the general population [20,92,97]. The mechanism through which this skewed protein intake distribution reduces weight loss remains unclear. Nonetheless, it is plausible that consuming protein intake exceeding the saturable dose estimate at any mealtime does not contribute to muscle synthesis [104]. Thus, varying protein intake throughout the day may result in increased oxidation during specific mealtimes where protein intake exceeds the saturable dose estimate, mimicking the effect of a low-protein diet. Because depleted muscle mass resulting from a low-protein diet is associated with lower resting energy expenditure [105], this condition may interfere with weight loss. Individuals participating in weight loss programs may also benefit from consuming breakfast as indicated by included cohort articles [66,72]. Although human trials suggest that skipping breakfast does not increase energy expenditure [34,75], it does lead to suppression of lipid oxidation [103], potentially increasing body fat storage. Thus, incorporating breakfast consumption into weight loss strategies, alongside increased physical activity or energy restriction, may synergistically promote lipid oxidation and subsequently reduce BW. Therefore, assuming the validity of these findings, the implementation of this time-related dietary intake may be beneficial in specific conditions in which its efficacy has been demonstrated. Nevertheless, further investigation is warranted to understand its broader applicability.

Because this study aims to isolate the impact of within-day dietary intake variability on health outcomes, interventions should maintain consistent energy and nutrient intake throughout the day, with meal timing being the sole variation. However, this consistency is not evident in several included randomized trials investigating the effect of TRE on adiposity because participants were allowed to eat ad libitum [22,36,54]. The effectiveness of TRE itself could be obscured by the fact that adopting TRE resulted in either lower TDEI [49] or lower than habitual intake [55]. Although an RCT involving obese participants observed greater weight loss with TRE compared to a control group under energy restriction [56], other trials involving nonobese participants that controlled energy intake in the control group failed to replicate this finding [29,58]. The question whether TRE only benefits individuals with obesity is subject to discussion. In a rodent study involving animals fed a high-fat diet, TRE resulted in improved circadian rhythmicity, suppressed lipogenesis, and reduced BW and BF compared to those on an ad libitum high-fat diet. However, these metabolic benefits were not observed in rodents fed a normal diet when subjected to TRE [104]. These findings suggest that, concerning BW management, it is still inconclusive whether TRE offers additional benefits beyond those of a traditional energy-restricted diet.

Discrepancies in outcomes may also be attributed to the amount of meal or energy intake allocated to a particular end of an eating window, either morning or evening. This observation falls under the temporal distribution category. Studies have demonstrated that allocating 45%–54% of TDEI to either end of the eating window did not affect BW in 2 randomized trials [96,101]. However, in 2 trials, allocating 70% or more of TDEI to the morning, which was almost similar to TRE but without strict eating window constraints, resulted in lower BW [32,100]. These findings suggest that human metabolism exhibits remarkable resilience to meal timing conditions, requiring an exceptionally high-energy intake to manifest adverse or beneficial metabolic outcomes, depending on the time allocation of energy intake. The underlying mechanisms of this phenomenon remain unclear, and it is uncertain whether the circadian rhythm plays a role. Mechanistic studies involving animal subjects showed that TRE can influence circadian clock in favor of reduced adiposity. However, the feeding scheme usually implemented by these studies is ad libitum [105,106], which may result in energy intake lower than the daily energy requirement and does not replicate the concentrated energy intake shown in the aforementioned studies [32,100].

This systematic review is subject to both strengths and limitations. We aimed to minimize bias, by involving more than one person to select titles for inclusion and in assessing the risk of bias. Although several excellent systematic reviews in this area have been conducted previously, most have focused on just one aspect only, such as TRE [107] or breakfast skipping [108]. The downside of this approach is that various intervention terms may refer to the same condition. For instance, dietary intake distribution skewed toward the morning may be referred to as breakfast omission, late TRE, or late caloric midpoint. In this systematic review, we therefore used generic keywords in the search strategy to encompass all possible terms related to variations in dietary intake. However, this approach resulted in numerous irrelevant studies to screen out and very low yield. Also, the drawback of using generic terms is that they may result in different article coverage compared to the use of specific keywords. A study suggested that only 7% of articles were common between reviews that utilized generic compared with specific keywords [109]. although we intended to cover energy and macronutrient intake, data on variation in carbohydrate and fat intake were limited, making between-study comparisons impossible in several categories.

Acquiring accurate data on the timing of individual food intake is crucial for capturing the intricate relationship between between-day and within-day dietary variation and health outcomes. The possibility of measurement error in self-reported dietary assessments cannot be overlooked, as it may introduce bias in the association between diet and health outcomes. Moreover, this issue may also necessitate larger sample sizes due to reduced statistical power [110]. Notably, several findings in our systematic review exhibited inconsistency, and whether this inconsistency is attributable to measurement error remains to be evaluated. Nevertheless, the necessity for stringent evaluations of dietary intake remains, now extending to include meal timing. However, this approach may impose a significant burden on study participants, potentially limiting its widespread adoption. As an alternative, researchers may consider utilizing questionnaires to inquire about the proportion of meal consumed at different times, providing an approximate assessment of habitual dietary intake temporal distribution. The primary challenge in assessing meal timing is determining which meals to focus on. Examining the timing of just one particular meal overlooks the timing of other eating occasions that collectively influence the daily temporal distribution of dietary intake. Therefore, we suggest that future studies adopt a holistic approach to meal timing assessment, taking into account the timing of all meals to accurately capture dietary intake throughout the day. Researchers might consider using wearable devices that provide real-time data on meal timing, such as continuous glucose monitors [111] or automatic ingestion monitors [112]. Moreover, a major challenge in dietary intervention trials is maintaining blinded treatment. Nevertheless, blinding interventions focused on meal timing or eating windows might be feasible in specific settings. For example, studies conducted in confinement, where participants lose access to time cues [113], could potentially blind participants to the timing of their meals. Finally, nutrition researchers could benefit from establishing a standardized term to describe variability in dietary intake presented in this systematic review, facilitating the identification of relevant studies by future systematic reviewers. “Time-related dietary variation” could be a potential candidate term.

Establishing causal links between any category of between- or within-day variation in dietary intake discussed in this systematic review is a challenging endeavor, given the available evidence. Nevertheless, the evidence available suggests that breakfast omission among healthy individuals may be the most compelling within-day variation in dietary intake associated with glucose homeostasis. Findings regarding other categories of dietary intake variations appear inconsistent, with associations with adiposity perceived to be context-dependent, such as in weight loss settings or in the presence of T2DM.

## Author contributions

The authors’ responsibilities were as follows – PS: designed the study and wrote the manuscript; PS, NP: performed literature search; PS, NP, JV: selected articles to be included; PS, NP: conducted risk of bias analysis; JV, EF: critically reviewed the manuscript; EF: finalized the manuscript; and all authors: read and approved the final manuscript.

## Funding

The preparation of this systematic review was supported financially by Indonesian Endowment Fund for Education (LPDP), the Ministry of Finance Republic of Indonesia. The sponsor had no involvement in the study design, collection, analysis, and interpretation of data, and writing and submission of the report.

## Data availability

Data used in this manuscript will be made available upon request pending application and approval.

## Conflict of interest

The authors report no conflicts of interest.
