# Supplementary material for: Associations between Variability in Between- and Within-Day Dietary Intake with Adiposity and Glucose Homeostasis in Adults: A Systematic Review
Source: Adv Nutr. 2024 Oct 9;15(11):100310. doi: 10.1016/j.advnut.2024.100310 (PMC11566682; doi:10.1016/j.advnut.2024.100310)
Supplement: Multimedia component 1 [file mmc1.docx]

**Supplementary Table 1**. Preferred Reporting Items for Systematic Reviews and Meta-Analyses (PRISMA) check list

| **Section and Topic** | **Item #** | **Checklist item** | **Location where item is reported** |
| --- | --- | --- | --- |
| **TITLE** | | |  |
| Title | 1 | Identify the report as a systematic review. | Page 1 |
| **ABSTRACT** | | |  |
| Abstract | 2 | See the PRISMA 2020 for Abstracts checklist. | Page 1-2 |
| **INTRODUCTION** | | |  |
| Rationale | 3 | Describe the rationale for the review in the context of existing knowledge. | Page 3-5 |
| Objectives | 4 | Provide an explicit statement of the objective(s) or question(s) the review addresses. | Page 5 |
| **METHODS** | | |  |
| Eligibility criteria | 5 | Specify the inclusion and exclusion criteria for the review and how studies were grouped for the syntheses. | Page 6 |
| Information sources | 6 | Specify all databases, registers, websites, organisations, reference lists and other sources searched or consulted to identify studies. Specify the date when each source was last searched or consulted. | Page 5 |
| Search strategy | 7 | Present the full search strategies for all databases, registers and websites, including any filters and limits used. | Table S3 |
| Selection process | 8 | Specify the methods used to decide whether a study met the inclusion criteria of the review, including how many reviewers screened each record and each report retrieved, whether they worked independently, and if applicable, details of automation tools used in the process. | Page 7 |
| Data collection process | 9 | Specify the methods used to collect data from reports, including how many reviewers collected data from each report, whether they worked independently, any processes for obtaining or confirming data from study investigators, and if applicable, details of automation tools used in the process. | Page 7 |
| Data items | 10a | List and define all outcomes for which data were sought. Specify whether all results that were compatible with each outcome domain in each study were sought (e.g. for all measures, time points, analyses), and if not, the methods used to decide which results to collect. | Page 6 |
|  | 10b | List and define all other variables for which data were sought (e.g. participant and intervention characteristics, funding sources). Describe any assumptions made about any missing or unclear information. | Page 6 |
| Study risk of bias assessment | 11 | Specify the methods used to assess risk of bias in the included studies, including details of the tool(s) used, how many reviewers assessed each study and whether they worked independently, and if applicable, details of automation tools used in the process. | Page 7-8 |
| Effect measures | 12 | Specify for each outcome the effect measure(s) (e.g. risk ratio, mean difference) used in the synthesis or presentation of results. | Page 8 |
| Synthesis methods | 13a | Describe the processes used to decide which studies were eligible for each synthesis (e.g. tabulating the study intervention characteristics and comparing against the planned groups for each synthesis (item #5)). | NA, all studies included |
|  | 13b | Describe any methods required to prepare the data for presentation or synthesis, such as handling of missing summary statistics, or data conversions. | NA |
|  | 13c | Describe any methods used to tabulate or visually display results of individual studies and syntheses. | Page 8 |
|  | 13d | Describe any methods used to synthesize results and provide a rationale for the choice(s). If meta-analysis was performed, describe the model(s), method(s) to identify the presence and extent of statistical heterogeneity, and software package(s) used. | Page 8 |
|  | 13e | Describe any methods used to explore possible causes of heterogeneity among study results (e.g. subgroup analysis, meta-regression). | NA |
|  | 13f | Describe any sensitivity analyses conducted to assess robustness of the synthesized results. | NA |
| Reporting bias assessment | 14 | Describe any methods used to assess risk of bias due to missing results in a synthesis (arising from reporting biases). | Page 7 |
| Certainty assessment | 15 | Describe any methods used to assess certainty (or confidence) in the body of evidence for an outcome. | NA |
| **RESULTS** | | |  |
| Study selection | 16a | Describe the results of the search and selection process, from the number of records identified in the search to the number of studies included in the review, ideally using a flow diagram. | Page 9 & Fig.1 |
|  | 16b | Cite studies that might appear to meet the inclusion criteria, but which were excluded, and explain why they were excluded. | Page 9 & Fig.1 |
| Study characteristics | 17 | Cite each included study and present its characteristics. | Table1-7 |
| Risk of bias in studies | 18 | Present assessments of risk of bias for each included study. | Table1-7, Table S4-6 |
| Results of individual studies | 19 | For all outcomes, present, for each study: (a) summary statistics for each group (where appropriate) and (b) an effect estimate and its precision (e.g. confidence/credible interval), ideally using structured tables or plots. | Table1-7 |
| Results of syntheses | 20a | For each synthesis, briefly summarise the characteristics and risk of bias among contributing studies. | Each category in the result section |
|  | 20b | Present results of all statistical syntheses conducted. If meta-analysis was done, present for each the summary estimate and its precision (e.g. confidence/credible interval) and measures of statistical heterogeneity. If comparing groups, describe the direction of the effect. | NA |
|  | 20c | Present results of all investigations of possible causes of heterogeneity among study results. | NA |
|  | 20d | Present results of all sensitivity analyses conducted to assess the robustness of the synthesized results. | NA |
| Reporting biases | 21 | Present assessments of risk of bias due to missing results (arising from reporting biases) for each synthesis assessed. | Table S4-6 |
| Certainty of evidence | 22 | Present assessments of certainty (or confidence) in the body of evidence for each outcome assessed. | NA |
| **DISCUSSION** | | |  |
| Discussion | 23a | Provide a general interpretation of the results in the context of other evidence. | Page 29-32 |
|  | 23b | Discuss any limitations of the evidence included in the review. | Page 32-33 |
|  | 23c | Discuss any limitations of the review processes used. | Page 32-33 |
|  | 23d | Discuss implications of the results for practice, policy, and future research. | Page 33 |
| **OTHER INFORMATION** | | |  |
| Registration and protocol | 24a | Provide registration information for the review, including register name and registration number, or state that the review was not registered. | Page 3 |
|  | 24b | Indicate where the review protocol can be accessed, or state that a protocol was not prepared. | Page 3 |
|  | 24c | Describe and explain any amendments to information provided at registration or in the protocol. | Page 5 |
| Support | 25 | Describe sources of financial or non-financial support for the review, and the role of the funders or sponsors in the review. | Page 34-35 |
| Competing interests | 26 | Declare any competing interests of review authors. | Page 34 |
| Availability of data, code and other materials | 27 | Report which of the following are publicly available and where they can be found: template data collection forms; data extracted from included studies; data used for all analyses; analytic code; any other materials used in the review. | Page 35 |

**Supplementary Table 2**. Preferred Reporting Items for Systematic Reviews and Meta-Analyses (PRISMA) abstract check list

| **Section and Topic** | **Item #** | **Checklist item** | **Reported (Yes/No)** |
| --- | --- | --- | --- |
| **TITLE** | | |  |
| Title | 1 | Identify the report as a systematic review. | Yes |
| **BACKGROUND** | | |  |
| Objectives | 2 | Provide an explicit statement of the main objective(s) or question(s) the review addresses. | Yes |
| **METHODS** | | |  |
| Eligibility criteria | 3 | Specify the inclusion and exclusion criteria for the review. | Yes |
| Information sources | 4 | Specify the information sources (e.g. databases, registers) used to identify studies and the date when each was last searched. | Yes |
| Risk of bias | 5 | Specify the methods used to assess risk of bias in the included studies. | No |
| Synthesis of results | 6 | Specify the methods used to present and synthesise results. | Yes |
| **RESULTS** | | |  |
| Included studies | 7 | Give the total number of included studies and participants and summarise relevant characteristics of studies. | Yes |
| Synthesis of results | 8 | Present results for main outcomes, preferably indicating the number of included studies and participants for each. If meta-analysis was done, report the summary estimate and confidence/credible interval. If comparing groups, indicate the direction of the effect (i.e. which group is favoured). | Yes |
| **DISCUSSION** | | |  |
| Limitations of evidence | 9 | Provide a brief summary of the limitations of the evidence included in the review (e.g. study risk of bias, inconsistency and imprecision). | Yes |
| Interpretation | 10 | Provide a general interpretation of the results and important implications. | Yes |
| **OTHER** | | |  |
| Funding | 11 | Specify the primary source of funding for the review. | Yes |
| Registration | 12 | Provide the register name and registration number. | Yes |

**Supplementary Table 3**. Search strategies implemented in each literature database

| Database | Keywords |
| --- | --- |
| PUBMED | (("variation*"[Title/Abstract] OR "dissimilarit*"[Title/Abstract] OR "similarit*"[Title/Abstract] OR "evenness"[Title/Abstract] OR "variabilit*"[Title/Abstract] OR "fluctuation*"[Title/Abstract] OR "disequilibrium"[Title/Abstract] OR "inconsisten*"[Title/Abstract] OR "consisten*"[Title/Abstract] OR "irregular*"[Title/Abstract] OR "regular*"[Title/Abstract] OR "distribution*"[Title/Abstract]) AND ("dietary intake*"[Title/Abstract] OR "calorie intake*"[Title/Abstract] OR "energy intake*"[Title/Abstract] OR "food intake*"[Title/Abstract] OR "macronutrient intake*"[Title/Abstract] OR "carbohydrate intake*"[Title/Abstract] OR "protein intake*"[Title/Abstract] OR "fat intake*"[Title/Abstract] OR "nutrient intake*"[Title/Abstract]) AND ("overweight"[Title/Abstract] OR "obesity"[Title/Abstract] OR "obese"[Title/Abstract] OR "body mass index"[Title/Abstract] OR "BMI"[Title/Abstract] OR "body fat"[Title/Abstract] OR "body fatness"[Title/Abstract] OR "fat percentage"[Title/Abstract] OR "body composition"[Title/Abstract] OR "HbA1c"[Title/Abstract] OR "fasting glucose"[Title/Abstract] OR "fasting blood glucose"[Title/Abstract] OR "fasting plasma glucose"[Title/Abstract] OR "fasting serum glucose"[Title/Abstract] OR "post-prandial glucose"[Title/Abstract] OR "glucose concentration"[Title/Abstract] OR "glucose response"[Title/Abstract] OR "glucose area under the curve"[Title/Abstract] OR "glucose excursion"[Title/Abstract] OR "glucose tolerance"[Title/Abstract] OR "glucose intolerance"[Title/Abstract] OR "glucose level"[Title/Abstract] OR "incremental AUC"[Title/Abstract] OR "total AUC"[Title/Abstract] OR "net AUC"[Title/Abstract] OR "glucose disposal"[Title/Abstract] OR "glucose clearance"[Title/Abstract] OR "glucose elimination"[Title/Abstract] OR "glucose disappearance"[Title/Abstract] OR "glucose uptake"[Title/Abstract] OR "insulin sensitivity"[Title/Abstract] OR "insulin resistance"[Title/Abstract] OR "insulin level"[Title/Abstract] OR "insulin concentration"[Title/Abstract] OR "fasting insulin"[Title/Abstract] OR "serum insulin"[Title/Abstract] OR "plasma insulin"[Title/Abstract] OR "reciprocal insulin"[Title/Abstract] OR "fasting glucose insulin ratio"[Title/Abstract] OR "FGIR"[Title/Abstract] OR "QUICKI"[Title/Abstract] OR "McAuley index"[Title/Abstract] OR "Matsuda index"[Title/Abstract] OR "Belfiore index"[Title/Abstract] OR "Cederholm index"[Title/Abstract] OR "Avignon index"[Title/Abstract] OR "Stumvoll index"[Title/Abstract] OR "Gutt index"[Title/Abstract] OR "glucose clamp"[Title/Abstract] OR "euglycemic clamp"[Title/Abstract] OR "hyperinsulinemic clamp"[Title/Abstract] OR "insulinemic clamp"[Title/Abstract] OR "insulin-suppression test"[Title/Abstract] OR "insulin infusion rate"[Title/Abstract])) NOT ("rat"[Title/Abstract] OR "rats"[Title/Abstract] OR "mouse"[Title/Abstract] OR "mice"[Title/Abstract] OR "rabbit*"[Title/Abstract] OR "guinea pig*"[Title/Abstract] OR "pig"[Title/Abstract] OR "pigs"[Title/Abstract] OR "swine*"[Title/Abstract] OR "gerbil*"[Title/Abstract] OR "hamster*"[Title/Abstract] OR "armadillo*"[Title/Abstract] OR "chinchilla*"[Title/Abstract] OR "bird"[Title/Abstract] OR "birds"[Title/Abstract] OR "avian*"[Title/Abstract] OR "zebrafish*"[Title/Abstract]) |
| SCOPUS | ( TITLE-ABS ( variation* OR dissimilarit* OR similarit* OR evenness OR variabilit* OR fluctuation* OR disequilibrium OR inconsisten* OR consisten* OR irregular* OR regular* OR distribution* ) ) AND ( TITLE-ABS ( "dietary intake*" OR "calorie intake*" OR "energy intake*" OR "food intake*" OR "macronutrient intake*" OR "carbohydrate intake*" OR "protein intake*" OR "fat intake*" OR "nutrient intake*" ) ) AND ( TITLE-ABS ( overweight OR obesity OR obese OR "body mass index" OR bmi OR "body fat" OR "body fatness" OR "fat percentage" OR "body composition" OR "HbA1c" OR "fasting glucose" OR "fasting blood glucose" OR "fasting plasma glucose" OR "fasting serum glucose" OR "post-prandial glucose" OR "glucose concentration" OR "glucose response" OR "glucose area under the curve" OR "glucose excursion" OR "glucose tolerance" OR "glucose intolerance" OR "glucose level" OR "incremental AUC" OR "total AUC" OR "net AUC" OR "glucose disposal" OR "glucose clearance" OR "glucose elimination" OR "glucose disappearance" OR "glucose uptake" OR "insulin sensitivity" OR "insulin resistance" OR "insulin level" OR "insulin concentration" OR "fasting insulin" OR "serum insulin" OR "plasma insulin" OR "reciprocal insulin" OR "fasting glucose insulin ratio" OR "FGIR" OR "QUICKI" OR "McAuley index" OR "Matsuda index" OR "Belfiore index" OR "Cederholm index" OR "Avignon index" OR "Stumvoll index" OR "Gutt index" OR "glucose clamp" OR "euglycemic clamp" OR "hyperinsulinemic clamp" OR "insulinemic clamp" OR "insulin-suppression test" OR "insulin infusion rate" ) ) AND NOT ( TITLE-ABS ( "rat" OR "rats" OR "mouse" OR "mice" OR "rabbit*" OR "guinea pig*" OR "pig" OR "pigs" OR "swine*" OR "gerbil*" OR "hamster*" OR "armadillo*" OR "chinchilla*" OR "bird" OR "birds" OR "avian*" OR "zebrafish*" ) ) |
| COCHRANE LIBRARY | (variation OR dissimilarit* OR similarit* OR evenness OR variabilit* OR fluctuation* OR disequilibrium OR inconsistenc* OR consistenc* OR inconsistent OR consistent OR irregularit* OR regularit* OR irregular OR regular OR distribution*):ti,ab,kw AND (“dietary intake“ OR “calorie intake“ OR “energy intake” OR “food intake“ OR “macronutrient intake“ OR “carbohydrate intake“ OR “protein intake“ OR “fat intake“ OR “nutrient intake” OR “dietary intakes“ OR “calorie intakes“ OR “energy intakes” OR “food intakes“ OR “macronutrient intakes“ OR “carbohydrate intakes“ OR “protein intakes“ OR “fat intakes“ OR “nutrient intakes”):ti,ab,kw AND (overweight OR obesity OR obese OR “body mass index” OR BMI OR “body fat” OR “body fatness” OR “fat percentage” OR “body composition” OR "HbA1c" OR "fasting glucose" OR "fasting blood glucose" OR "fasting plasma glucose" OR "fasting serum glucose" OR "post-prandial glucose" OR "glucose concentration" OR "glucose response" OR "glucose area under the curve" OR "glucose excursion" OR "glucose tolerance" OR "glucose intolerance" OR "glucose level" OR "incremental AUC" OR "total AUC" OR "net AUC" OR "glucose disposal" OR "glucose clearance" OR "glucose elimination" OR "glucose disappearance" OR "glucose uptake" OR "insulin sensitivity" OR "insulin resistance" OR "insulin level" OR "insulin concentration" OR "fasting insulin" OR "serum insulin" OR "plasma insulin" OR "reciprocal insulin" OR "fasting glucose insulin ratio" OR "FGIR" OR "QUICKI" OR "McAuley index" OR "Matsuda index" OR "Belfiore index" OR "Cederholm index" OR "Avignon index" OR "Stumvoll index" OR "Gutt index" OR "glucose clamp" OR "euglycemic clamp" OR "hyperinsulinemic clamp" OR "insulinemic clamp" OR "insulin-suppression test" OR "insulin infusion rate"):ti,ab,kw NOT(rat OR rats OR mouse OR mice OR rabbit* OR guinea pig* OR pig OR pigs OR swine* OR gerbil* OR hamster* OR armadillo* OR chinchilla* OR bird OR birds OR avian* OR zebrafish*):ti,ab,kw |
| OPEN GREY | abstract:(variation OR dissimilarit* OR similarit* OR evenness OR variabilit* OR fluctuation* OR disequilibrium OR inconsistenc* OR consistenc* OR inconsistent OR consistent OR irregularit* OR regularit* OR irregular OR regular OR distribution*) AND abstract:(“dietary intake“ OR “calorie intake“ OR “energy intake” OR “food intake“ OR “macronutrient intake“ OR “carbohydrate intake“ OR “protein intake“ OR “fat intake“ OR “nutrient intake” OR “dietary intakes“ OR “calorie intakes“ OR “energy intakes” OR “food intakes“ OR “macronutrient intakes“ OR “carbohydrate intakes“ OR “protein intakes“ OR “fat intakes“ OR “nutrient intakes”) AND abstract:(overweight OR obesity OR obese OR “body mass index” OR BMI OR “body fat” OR “body fatness” OR “fat percentage” OR “body composition” OR "HbA1c" OR "fasting glucose" OR "fasting blood glucose" OR "fasting plasma glucose" OR "fasting serum glucose" OR "post-prandial glucose" OR "glucose concentration" OR "glucose response" OR "glucose area under the curve" OR "glucose excursion" OR "glucose tolerance" OR "glucose intolerance" OR "glucose level" OR "incremental AUC" OR "total AUC" OR "net AUC" OR "glucose disposal" OR "glucose clearance" OR "glucose elimination" OR "glucose disappearance" OR "glucose uptake" OR "insulin sensitivity" OR "insulin resistance" OR "insulin level" OR "insulin concentration" OR "fasting insulin" OR "serum insulin" OR "plasma insulin" OR "reciprocal insulin" OR "fasting glucose insulin ratio" OR "FGIR" OR "QUICKI" OR "McAuley index" OR "Matsuda index" OR "Belfiore index" OR "Cederholm index" OR "Avignon index" OR "Stumvoll index" OR "Gutt index" OR "glucose clamp" OR "euglycemic clamp" OR "hyperinsulinemic clamp" OR "insulinemic clamp" OR "insulin-suppression test" OR "insulin infusion rate") NOT abstract:("rat" OR "rats" OR "mouse" OR "mice" OR "rabbit*" OR "guinea pig*" OR "pig" OR "pigs" OR "swine*" OR "gerbil*" OR "hamster*" OR "armadillo*" OR "chinchilla*" OR "bird" OR "birds" OR "avian*" OR "zebrafish*") |

**Supplementary Table 4**. NHLBI Quality Assessment Tool for Cohort and Cross-Sectional Studies

| Author(s) | RQ | SP | PR | S | SS | EM | TF | EL | EC | EA | OM | OB | FL | CON | OV |
| --- | --- | --- | --- | --- | --- | --- | --- | --- | --- | --- | --- | --- | --- | --- | --- |
| Azuma, et al, 2023 | 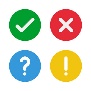 | 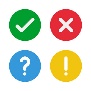 | 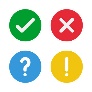 | 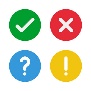 | 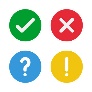 | 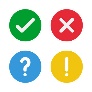 | 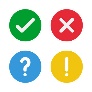 | 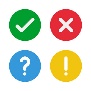 | 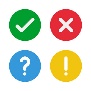 | 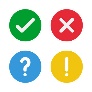 | 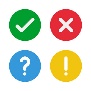 | 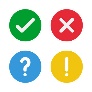 | 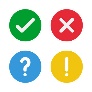 | 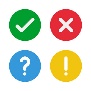 | 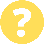 |
| Batista-Jorge, et al, 2016 | 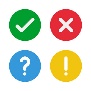 | 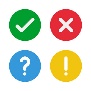 | 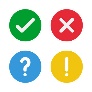 | 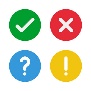 | 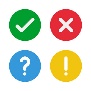 | 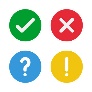 | 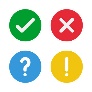 | 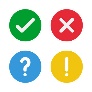 | 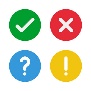 | 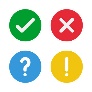 | 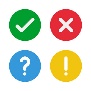 | 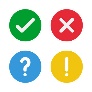 | 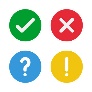 | 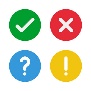 | 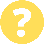 |
| Dattilo, et al, 2011 | 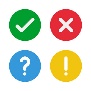 | 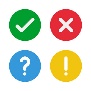 | 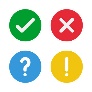 | 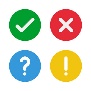 | 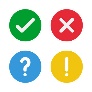 | 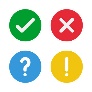 | 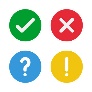 | 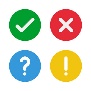 | 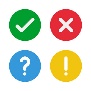 | 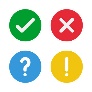 | 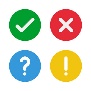 | 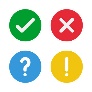 | 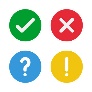 | 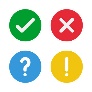 | 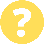 |
| Ding, et al, 2020 | 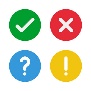 | 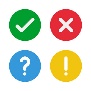 | 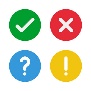 | 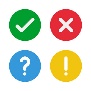 | 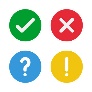 | 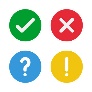 | 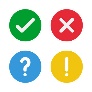 | 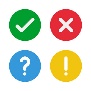 | 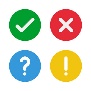 | 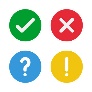 | 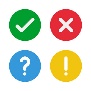 | 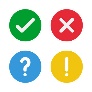 | 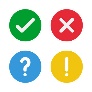 | 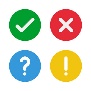 | 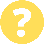 |
| Dote-Montero, et al, 2023 | 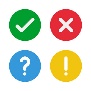 | 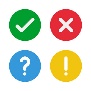 | 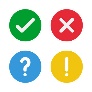 | 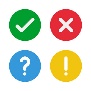 | 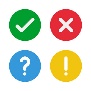 | 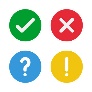 | 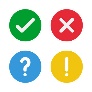 | 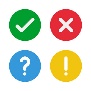 | 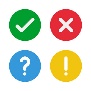 | 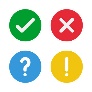 | 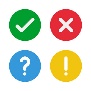 | 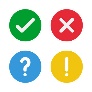 | 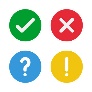 | 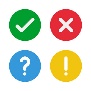 | 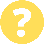 |
| Elahy, et al, 2023 | 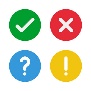 | 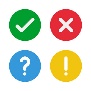 | 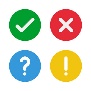 | 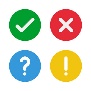 | 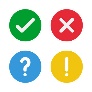 | 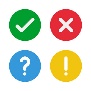 | 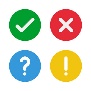 | 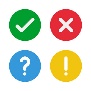 | 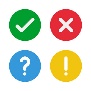 | 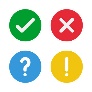 | 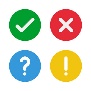 | 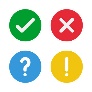 | 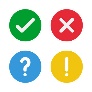 | 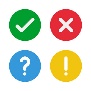 | 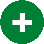 |
| Eom, et al, 2022 | 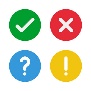 | 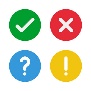 | 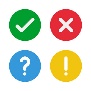 | 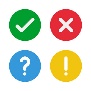 | 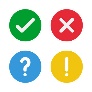 | 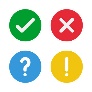 | 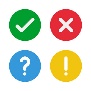 | 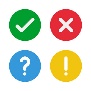 | 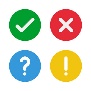 | 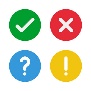 |  |  |  |  |  |
| Farsijani, et al, 2016 |  |  |  |  |  |  |  |  |  |  |  |  |  |  |  |
| Farsijani, et al, 2020 |  |  |  |  |  |  |  |  |  |  |  |  |  |  |  |
| Fleischer, et al, 2022 |  |  |  |  |  |  |  |  |  |  |  |  |  |  |  |
| Forester, et al, 2018 |  |  |  |  |  |  |  |  |  |  |  |  |  |  |  |
| Garaulet, et al, 2013 |  |  |  |  |  |  |  |  |  |  |  |  |  |  |  |
| Garaulet, et al, 2016 |  |  |  |  |  |  |  |  |  |  |  |  |  |  |  |
| Hashimoto, et al, 2020 |  |  |  |  |  |  |  |  |  |  |  |  |  |  |  |
| Helo, et al, 2021 |  |  |  |  |  |  |  |  |  |  |  |  |  |  |  |
| Hermenegildo, et al, 2016 |  |  |  |  |  |  |  |  |  |  |  |  |  |  |  |
| Horn, et al, 2022 |  |  |  |  |  |  |  |  |  |  |  |  |  |  |  |
| Hunt, et al, 2020 |  |  |  |  |  |  |  |  |  |  |  |  |  |  |  |
| Jacob, et al, 2020 |  |  |  |  |  |  |  |  |  |  |  |  |  |  |  |
| Jacob, et al, 2023 |  |  |  |  |  |  |  |  |  |  |  |  |  |  |  |
| Jayedi, et al, 2020 |  |  |  |  |  |  |  |  |  |  |  |  |  |  |  |
| Kong, et al, 2012 |  |  |  |  |  |  |  |  |  |  |  |  |  |  |  |
| Leech, et al, 2017 |  |  |  |  |  |  |  |  |  |  |  |  |  |  |  |
| Ma, et al, 2003 |  |  |  |  |  |  |  |  |  |  |  |  |  |  |  |
| McCurley, et al, 2022 |  |  |  |  |  |  |  |  |  |  |  |  |  |  |  |
| McHill, et al, 2017 |  |  |  |  |  |  |  |  |  |  |  |  |  |  |  |
| Meule, et al, 2014 |  |  |  |  |  |  |  |  |  |  |  |  |  |  |  |
| Pallangyo, et al, 2020 |  |  |  |  |  |  |  |  |  |  |  |  |  |  |  |
| Park, et al, 2020 |  |  |  |  |  |  |  |  |  |  |  |  |  |  |  |
| Pot, Hardy, and Stephen, 2016 |  |  |  |  |  |  |  |  |  |  |  |  |  |  |  |
| Quist, et al, 2021 |  |  |  |  |  |  |  |  |  |  |  |  |  |  |  |
| Saintila, et al, 2023 |  |  |  |  |  |  |  |  |  |  |  |  |  |  |  |
| Saneei, et al, 2016 |  |  |  |  |  |  |  |  |  |  |  |  |  |  |  |
| Song, et al, 2005 |  |  |  |  |  |  |  |  |  |  |  |  |  |  |  |
| Takase, et al, 2019 |  |  |  |  |  |  |  |  |  |  |  |  |  |  |  |
| Teixeira, et al, 2019 |  |  |  |  |  |  |  |  |  |  |  |  |  |  |  |
| Terada, et al, 2019 |  |  |  |  |  |  |  |  |  |  |  |  |  |  |  |
| Widaman, et al, 2016 |  |  |  |  |  |  |  |  |  |  |  |  |  |  |  |
| Xiao, Garaulet, and Scheer, 2019 |  |  |  |  |  |  |  |  |  |  |  |  |  |  |  |
| Yoon, Choi, and Kim, 2021 |  |  |  |  |  |  |  |  |  |  |  |  |  |  |  |
| Yoshimura, et al, 2023 |  |  |  |  |  |  |  |  |  |  |  |  |  |  |  |
| Zerón-Rugerio, et al, 2019 |  |  |  |  |  |  |  |  |  |  |  |  |  |  |  |
| Zhao, et al, 2022 |  |  |  |  |  |  |  |  |  |  |  |  |  |  |  |

Yes; No; Cannot Determined, Not Applicable, Not Reported; Good; Fair; Poor

RQ: Was the research question or objective in this paper clearly stated?

SP: Was the study population clearly specified and defined?

PR: Was the participation rate of eligible persons at least 50%?

S: Were all the subjects selected or recruited from the same or similar populations (including the same time period)? Were inclusion and exclusion criteria for being in the study prespecified and applied uniformly to all participants?

SS: Was a sample size justification, power description, or variance and effect estimates provided?

EM: For the analyses in this paper, were the exposure(s) of interest measured prior to the outcome(s) being measured?

TF: Was the timeframe sufficient so that one could reasonably expect to see an association between exposure and outcome if it existed?

EL: For exposures that can vary in amount or level, did the study examine different levels of the exposure as related to the outcome (e.g., categories of exposure, or exposure measured as continuous variable)?

EC: Were the exposure measures (independent variables) clearly defined, valid, reliable, and implemented consistently across all study participants?

EA: Was the exposure(s) assessed more than once over time?

OM: Were the outcome measures (dependent variables) clearly defined, valid, reliable, and implemented consistently across all study participants?

OB: Were the outcome assessors blinded to the exposure status of participants?

FL: Was loss to follow-up after baseline 20% or less?

C: Were key potential confounding variables measured and adjusted statistically for their impact on the relationship between exposure(s) and outcome(s)?

OV: Overall risk of bias

**Supplementary Table 5**. The Risk of Bias In Non-randomized Studies – of Interventions (ROBINS-I) assessment tool

| Author(s) | C | S | CI | DI | M | OM | SR | OV |
| --- | --- | --- | --- | --- | --- | --- | --- | --- |
| Gill and Panda, 2015 |  |  |  |  |  |  |  |  |
| Jones, et al, 2020 |  |  |  |  |  |  |  |  |
| Kirkham, et al, 2023 |  |  |  |  |  |  |  |  |
| Parr, et al, 2020 |  |  |  |  |  |  |  |  |
| Parr, et al, 2023 |  |  |  |  |  |  |  |  |
| Wilkinson, et al, 2020 |  |  |  |  |  |  |  |  |
| Zhao, et al, 2022 |  |  |  |  |  |  |  |  |

Low; Moderate; Serious; Critical

C: Bias due to confounding

S: Bias in selection of participants into the study

CI: Bias in classification of interventions

DI: Bias due to deviations from intended interventions

M: Bias due to missing data

OM: Bias in measurement of outcomes

SR: Bias in selection of reported result

OV: Overall risk of bias

**Supplementary Table 6**. Cochrane risk-of-bias tool for randomized trials (RoB2)

| **Author(s)** | **R** | **DI** | **M** | **OM** | **SR** | **OV** |
| --- | --- | --- | --- | --- | --- | --- |
| Beaulieu, et al, 2020 |  |  |  |  |  |  |
| Beebe, et al, 1990 |  |  |  |  |  |  |
| Bowen, et al, 2018 |  |  |  |  |  |  |
| Chow, et al, 2020 |  |  |  |  |  |  |
| Chowdhury, et al, 2016 |  |  |  |  |  |  |
| Chowdhury, et al, 2018 |  |  |  |  |  |  |
| Correia, et al, 2021 |  |  |  |  |  |  |
| Hibi, et al, 2013 |  |  |  |  |  |  |
| Hudson, et al, 2017 |  |  |  |  |  |  |
| Jakubowicz, et al, 2013 |  |  |  |  |  |  |
| Keim, et al, 1997 |  |  |  |  |  |  |
| Kessler, et al, 2017 |  |  |  |  |  |  |
| Kim, et al, 2018 |  |  |  |  |  |  |
| Kobayashi, et al, 2014 |  |  |  |  |  |  |
| Kuwahara, et al, 2022 |  |  |  |  |  |  |
| Lombardo, et al, 2020 |  |  |  |  |  |  |
| Lowe, et al, 2020 |  |  |  |  |  |  |
| Martens, et al, 2020 |  |  |  |  |  |  |
| Moro, et al, 2021 |  |  |  |  |  |  |
| Nouripour, et al, 2021 |  |  |  |  |  |  |
| Ogata, et al, 2019 |  |  |  |  |  |  |
| Oliveira, et al, 2023 |  |  |  |  |  |  |
| Parkner, et al, 2011 |  |  |  |  |  |  |
| Parr, et al, 2018 |  |  |  |  |  |  |
| Ruddick-Collins, et al, 2022 |  |  |  |  |  |  |
| Singh, et al, 2020 |  |  |  |  |  |  |
| Steger, et al, 2023 |  |  |  |  |  |  |
| Vujovic, et al, 2022 |  |  |  |  |  |  |
| Yasuda, et al, 2020 |  |  |  |  |  |  |
| Zaman, et al, 2023 |  |  |  |  |  |  |

Low; Some concerns; High

R: Risk of bias arising from the randomization process

DI: Risk of bias due to deviation from the intended interventions (effect of assignment to intervention)

M: Missing outcome data

OM: Risk of bias in measurement of the outcome

SR: Risk of bias in selection of the reported result

OV: Overall risk of bias
